# CD30 as a Target Molecule in the Diagnosis and Therapy of Lymphomas

**DOI:** 10.1002/ajh.70107

**Published:** 2025-10-22

**Authors:** Harald Stein, Brunangelo Falini

**Affiliations:** ^1^ Past Berlin Reference Pathologist of the German Reference Center for Hematopathology Berlin Germany; ^2^ Institute of Hematology and Center for Hemato‐Oncological Research (CREO), University of Perugia and Santa Maria Della Misericordia Hospital Perugia Italy

**Keywords:** antibodies, brentuximab, CAR‐T cells, CD30, Hodgkin, immunohistochemistry, lymphomas

## Abstract

The tumor necrosis factor (TNF)‐receptor superfamily 8 receptor CD30 molecule is expressed in all tumor cells of Hodgkin lymphoma and anaplastic large cell lymphoma but is only weakly expressed in a small subset of large lymphoid cells in normal peripheral lymphoid tissues. This makes this molecule an important target for the diagnosis and treatment of CD30‐expressing lymphomas. We describe the road to the discovery of the CD30 molecule and the way CD30 has contributed to more precise diagnosis and classification of lymphomas. Moreover, we address how anti‐CD30 immunotherapy was developed and the impact of the anti‐CD30‐auristatin conjugate and anti‐CD30 CAR‐T cells in treating CD30‐expressing lymphomas.

## 1 Introduction

The dominant and strong expression of the TNF Receptor superfamily 8 receptor CD30 by the tumor cells of many lymphoma entities and the minor and low CD30 expression by normal cells in lymphoid tissues has made this molecule an important target for the diagnosis and treatment of lymphomas expressing CD30.

The introduction of CD30 into immunohistochemical diagnostic pathology has led to the definition of anaplastic large cell lymphoma (ALCL) as an entity, to a more precise subtyping of peripheral T‐cell lymphoma (PTCL), to the elucidation of the cellular origin of Hodgkin and Reed‐Sternberg (HRS) cells, and to the understanding of their special features. Moreover, it allowed a clearer differential diagnosis between classic Hodgkin lymphoma (cHL), ALCL with their subtypes, and mediastinal gray zone lymphoma. The precise detection of CD30 in routine biopsies has significantly contributed to the identification of lymphomas suitable for anti‐CD30 immunotherapy.

In the first part of this review, we describe the road to the discovery of the CD30 molecule and the way CD30 has contributed to more precise diagnosis and classification of lymphomas. In the second part of the review, we address how anti‐CD30 therapy was developed and the impact of the anti‐CD30–auristatin conjugate and anti‐CD30 CAR‐T cells in the therapy of CD30‐expressing lymphomas.

## 2 The Discovery of CD30

### 2.1 Identification of Many Large Cell Lymphomas as IgM‐Positive B‐Cell Lymhomas (1972)

In the 1960's, large cell neoplasms of lymph nodes were regarded as being derived from reticulum cells or from histiocytes by lymphoma experts in the USA and Europe [1, 2]. This view was mainly based on the dogma that lymphocytes do not have the capacity to transform into large cells. In 1965, immunological studies disclosed that lymphocytes can transform into large cells and consist of B‐ and T‐cells [3]. These discoveries were ignored by the lymphoma experts at that time.

In view of the observation that murine B cells carry on their surface immunoglobulin [4] one of the authors of this review (H.S.) measured IgM in fresh autopsy samples of reticulosarcomas and histiocytic lymphomas. Notably, high amounts of IgM were detected in these tumors, indicating that they were derived from B cells and represented large B‐cell lymphomas of high‐grade malignancy [5, 6].

Subsequent studies aimed at the identification of the cell of origin of the IgM‐negative large cell lymphomas, including the IgM‐negative HRS cells of cHL, were unsuccessful for a long time.

### 2.2 Establishment of the First Hodgkin Cell Line (1979)

**FIGURE 1:**
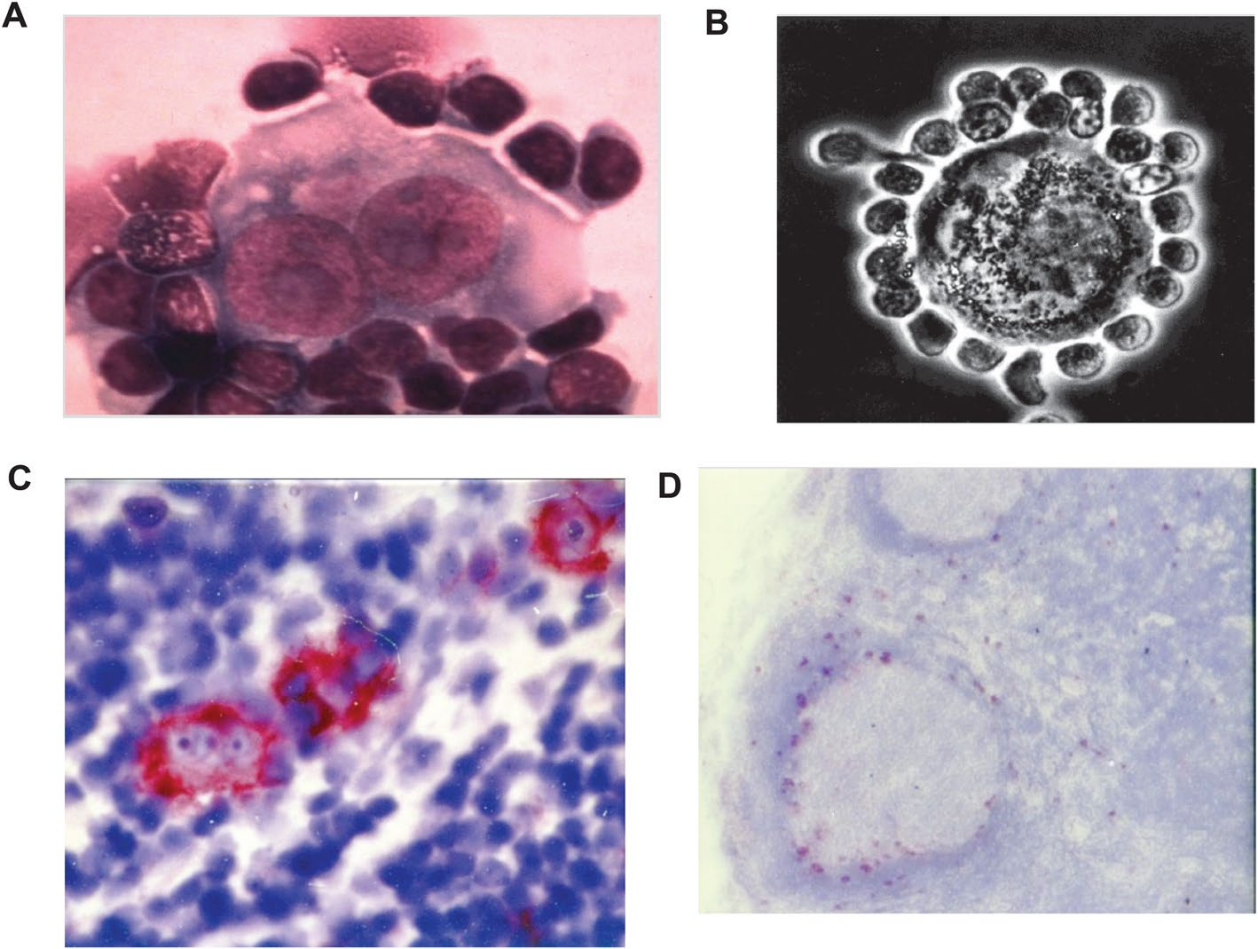
Recognition of L428 as a true Hodgkin cell line and generation of polyclonal and monoclonal antibodies to the L428 cell line cells. (A) Imprint of an in vitro Reed‐Sternberg cell surrounded by lymphocytes; (B) Co‐culture of the L428 cell line cell with peripheral lymphocytes from one of the authors (H.S.) showing strong binding of the lymphocytes to the surface of a L428 cell line; (C) The first monoclonal anti‐L428 cell antibody called Ki‐1 showed the same selective staining of HRS cells as the polyclonal rabbit antiserum in a frozen section of a Hodgkin biopsy and in addition (D) a weak staining of few normal lymphoid cells in a frozen section of a human tonsil. Figure 1 was previously published in Stein H and Diehl V, *Hematology*/*Oncology Clinics of North America*,28:1–11, 2014. [Color figure can be viewed at wileyonlinelibrary.com]

Progress occurred when cell lines of HRS cells were established. However, the first attempts led to false and fraudulent results published in 1977 [7, 8, 9]. This was disclosed 3 years later [10]. In 1979, Diehl's team succeeded in establishing the permanently growing cell line L428 from the pleural effusion of a patient with Hodgkin's disease [11]. It was first shown that the L428 cells resemble HRS cells by the lack of B‐cell, T‐cell, and histiocyte antigens. The subsequent search for a specific or characteristic marker for HRS cells was prompted by the observation that in vivo HRS cells are often surrounded by lymphocytes (Figure 1A). Co‐cultivation experiments of the L428 cells with peripheral lymphocytes from one of the authors (H.S.) revealed that the L428 cells bind lymphocytes to their surface as HRS cells do in vivo (Figure 1B). These findings strongly suggested that the L428 represents a true HRS cell line.

### 2.3 Search for Hodgkin Related Antigens (1981)

Vaccinations of several rabbits with a highly purified cytoplasmic protein fraction from the L428 cells were performed. The antiserum of one of the rabbits stained selectively the cytoplasm of HRS cells, in frozen sections of a Hodgkin biopsy, following absorption with neutrophils and Daudi cell line cells [12]. These findings suggested that the polyclonal antiserum detects a Hodgkin‐specific molecule, perhaps of viral nature.

### 2.4 Discovery of the Ki‐1 Antigen (1982)

In view of the above‐mentioned possibility, the monoclonal antibody (mAb) technology—established by Cesar Milstein and George Köhler—was applied to generate a hybridoma producing an antibody of identical quality and unlimited quantity directed at the antigen detected by the polyclonal antiserum. Among the 1500 generated hybridomas, one hybridoma was identified whose secreted antibodies stained in frozen sections the cytoplasm of HRS cells strongly (Figure 1C) and in frozen sections of a human tonsil, few large cells around B‐cell follicles (Figure 1D) [13, 14]. The identified hybridoma was called Ki‐1 [13, 14].

### 2.5 Classification of Ki‐1 According to the Cluster of Differentiation (CD) as CD30 (1986)

To get the Ki‐1 antibody included in the CD classification it was necessary to have further antibodies with Ki‐1 specificity. In Berlin, eight new Ki‐1‐like hybridomas were generated. The antibodies of these new hybridomas were accepted by the Leucocyte Typing Workshop in Oxford 1986. The Ki‐1 hybridomas were included in the CD cluster classification under the designation CD30 [15].

### 2.6 Generation of the Ber‐H2 Antibody for the Detection of the Ki‐1/CD30 Molecule in Routine Paraffin Sections (1989)

Among the eight new Ki‐1 hybridomas, one hybridoma with the designation “Ber‐H2” was identified, whose secreted antibodies detected a formol‐resistant epitope of the Ki‐1/CD30 molecule and thus enabled a strong and specific staining of CD30 in routine paraffin sections [16]. With the world‐wide availability of the Ber‐H2 antibody, CD30 was introduced into the diagnostic immunohistologic tissue programs [17, 18].

### 2.7 Classification of the IgM‐Negative Large Cell Lymphomas as Ki‐1/CD30 Lymphomas (1994)

**FIGURE 2:**
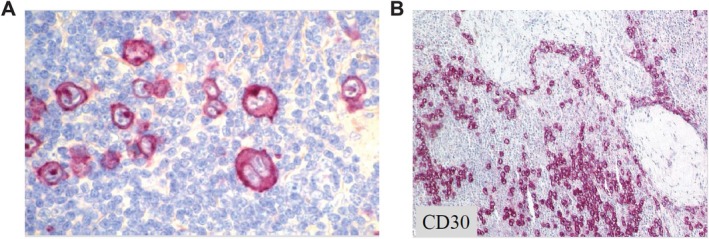
CD30 staining in formalin fixed and paraffin embedded (FFPE) tissue sections. Strong selective staining of HRS cells (A) and ALCL cells (B) by the monoclonal antibody Ber‐H2 in PPFE sections. Figures 2 a and b were previously published in Stein H and Diehl V, *Hematology*/*Oncology Clinics of North America*, 28:1–11, 2014. [Color figure can be viewed at wileyonlinelibrary.com]

Surprisingly, the Ki‐1 antibodies, besides HRS cells, also stained the IgM‐negative large cell lymphoma with anaplastic morphology [19, 20, 21], leading to the initial designation of these tumors as Ki‐1 lymphomas. The new anti‐CD30 antibody Ber‐H2 enabled the extension of the investigations about Ki‐1 lymphomas in routine paraffin sections (Figure 2A,B). The initial histologic classification of Ki‐1 lymphomas varied widely and included: malignant histiocytosis, anaplastic carcinoma, pleomorphic large cell lymphoma, and Hodgkin sarcoma. When these Ki‐1 positive cases were reviewed together, they appeared to belong to a single entity, designed as ALCL. This was accepted by the R.E.A.L [17] and WHO lymphoma classifications [18]. Subsequent immunoglobulin and T‐cell receptor (TCR) rearrangement studies identified Ki‐1/CD30‐positive tumors as a special T‐cell derived lymphoma entity with frequent loss of T‐cell program [22].

## 3 Features of the CD30 Molecule

### 3.1 Chromosomal Localization and Structure of the CD30 Molecule (1992)

**FIGURE 3:**
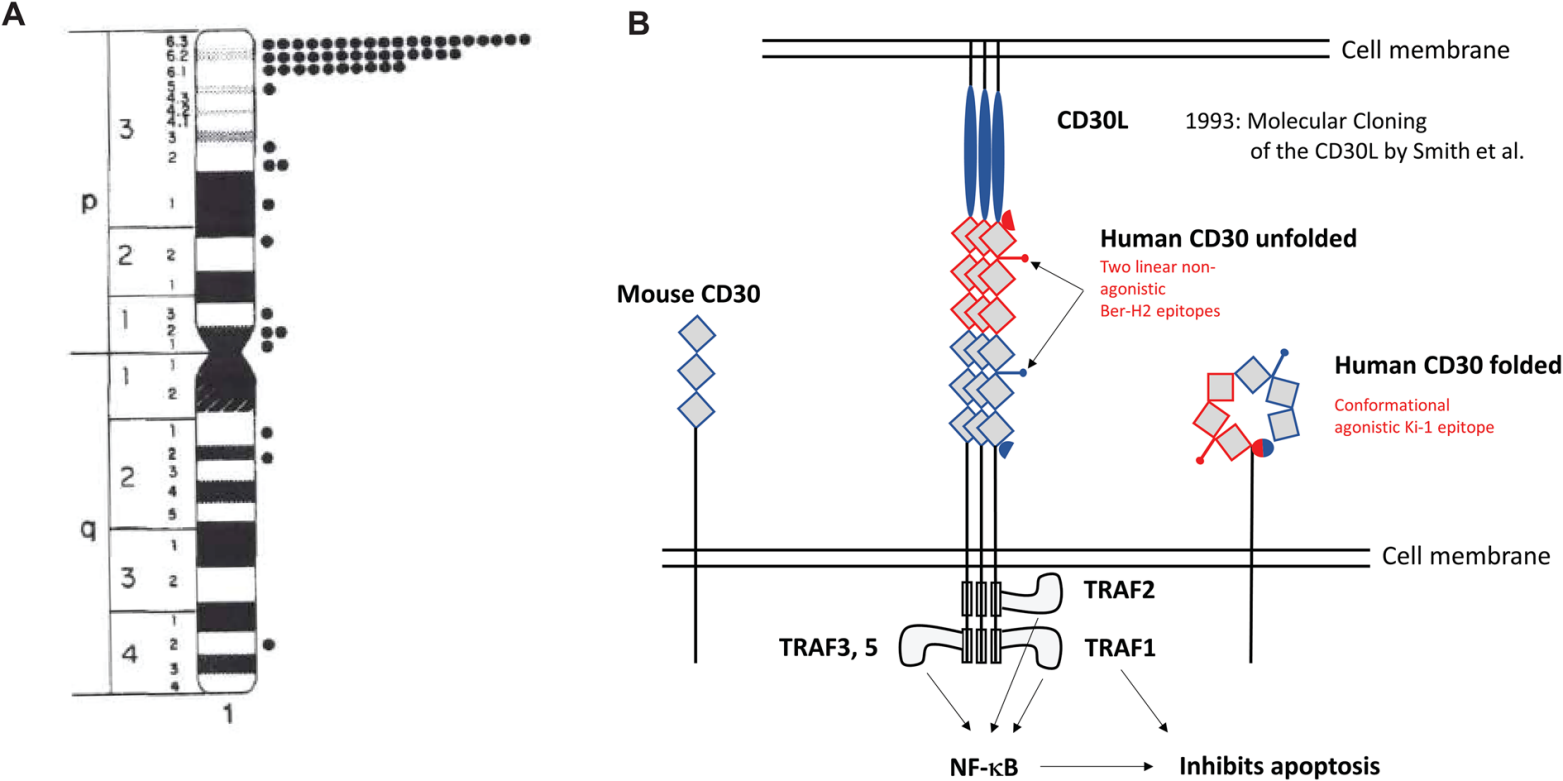
Molecular cloning of the Ki‐1/CD30 molecule. (Left) Chromosomal localisation of the Ki‐1/CD30; (Right) Structure of the Ki‐1/CD30 molecule. The extracellular non‐folded domain is comprised of six cystein repeats with two epitopes recognized by the Ber‐H2 antibody. The folded domain expresses an epitope to which the first Ki‐1 antibody is directed to. The intracellular domain contains TNF receptor associated factor binding sequences which can activate NF‐kB and inhibit apoptosis. [Color figure can be viewed at wileyonlinelibrary.com]

The CD30 gene is localized at chromosome 1p36.13 (Figure 3, left), closely linked to other members of the TNF receptor superfamily, such as the human TNFR2 and OX40 genes [23, 24] and represents a member of the Tumor Necrosis Factor Receptor (TNFR) superfamily [25, 26]. CD30 is a 120 kD glycoprotein with intracellular, transmembrane, and extracellular domains (Figure 3, right). The extracellular domain consists of six cysteine‐rich regions in a duplicated structure [25, 26, 27, 28]. All TNF family proteins, including CD30, form homotrimers, a configuration essential for their functionality. The cytoplasmic end of the CD30 protein contains TNF receptor associated factor binding sequences [27] that can activate the pathway of the nuclear factor kappa B (NFkB) transcription factor [29, 30]. The mature form of CD30 is processed from a precursor of about 84 kD during its passage through the Golgi complex [26]. The molecular‐weight shift from 84 to 120 kD is mostly due to glycosylation [31, 32]. The extracellular part of CD30 is cleaved proteolytically by a zinc metalloprotease (ADAM17). This soluble CD30 is released in the serum where it is detectable both in cHL [33] and ALCL [34] patients. One question is whether the soluble CD30 may bind and neutralize parts of the injected anti‐CD30 immunotoxin. However, reduction of therapeutic effect by the soluble CD30 appears unlikely since the immunotoxin is administered in excess as compared to the minimal amount of native anti‐CD30 monoclonal antibody that has been shown to target tumor cells in vivo [35].

### 3.2 Biological Function of the CD30 Protein

Epitope stimulation experiments of CD30 resulted in receptor trimerisation and signal transduction via recruitment of TNFR associated factors (TRAFs) and TRAF‐binding proteins, generating a signaling complex [36, 37, 38]. TRAF2, as well as TRAF1 and TRAF5, are all implicated in the signaling process. Downstream effects of CD30 stimulation are mediated in part by NFkB, as well as by mitogen‐activated protein kinases/extracellular signal‐regulated kinase pathways [29, 38, 39, 40, 41, 42, 43]. A novel domain in the CD30 cytoplasmic tail also mediates NFkB activation, without direct interaction of TRAF2 or 5, suggesting involvement of unknown TRAF protein(s) in the signal transduction pathway of CD30 [44].

The high and consistent expression of CD30 in cHL and ALCL and its rare and low expression in normal lymphoid tissues suggests that CD30 plays a significant role in the development of cHL and ALCL [45]. The finding that the NFkB and mitogen‐activated protein kinase/extracellular signal‐regulated kinase pathways are integral to CD30‐mediated signaling appears to be a hint that CD30 expression may confer a proliferative and anti‐apoptotic benefit in neoplastic cells [29, 45, 46]. Horie et al. [43] proposed a link between CD30 overexpression and ligand‐independent stimulation of the NFkB pathways in cHL cells, underscoring a possible link between CD30 expression and tumor perpetuation. The group of Stein et al. [45] could not replicate these findings and suggested that NFkB activation in cHL is constitutive and unrelated to CD30 but present in ALCL cells. Watanabe et al. [47] showed CD30 upregulation in cHL and ALCL cell lines might be linked by a self‐perpetuating loop through the mitogen‐activated protein kinase/extracellular signal‐regulated kinase pathway to the expression of JunB, a member of the activator protein (AP‐1) transcription factor family, with diverse effects including a possible link to malignant transformation. It was shown in ALCL cell lines that the transcription factor interferon regulatory factor 4 (IRF4) drives CD30 expression in a positive feedback loop involving NFkB [48]. In addition to IRF4 and AP‐1/JunB, the Ets transcription family has been implicated in tumor cell upregulation of CD30 [48, 49, 50, 51].

Additional studies sought to define the role of CD30 stimulation in lymphoma pathogenesis but were hampered by the application of differing ligands and different cell lines. The interpretation of the different studies and the pleiotropic effects of CD30 stimulation resulted in a remarkable degree of controversy. Thus, it is ultimately unclear whether CD30 contributes to the pathogenesis of CD30 expressing lymphomas.

## 4 Features of CD30‐Positive HRS Cells of cHL

### 4.1 Frequency of HRS Cells

When first described, the frequency of HRS cells assessed in hematoxylin–eosin stained sections of diseased tissue was 0.5%–2% [52, 53]. Mononuclear Hodgkin cells were often not recognized histologically because their morphology resembled that of other non‐malignant large cells. The low frequency figures stem from the time before the availability of the CD30 immunostaining. The inclusion of CD30 into the diagnostic programs showed that the frequency of HRS cells can reach values of > 10% in Hodgkin tissue biopsies (Stein H, unpublished findings).

### 4.2 Elucidation of Cellular Origin of HRS Cells by Picking CD30‐Positive Cells From Frozen Sections of Hodgkin Biopsies

The many studies to clarify the cellular origin of HRS cells failed because of the rareness of the HRS cells in the Hodgkin‐affected tissues. CD30 immunostaining made single HRS cells visible for their extraction from frozen sections. The isolated CD30‐positive HRS cells were subjected to single‐cell PCR and proved to contain clonally rearranged *IG* in the *V* genes, a hallmark of malignant B‐cells [52, 54, 55, 56]. High load of somatic mutations was found in the clonally rearranged V_H_ genes of HRS cells, suggesting that HRS cells are derived from germinal center B cells [52]. Unlike other B cell antigens, one molecule of the B‐cell program, PAX5, was not switched off. Its expression was a phenotypical confirmation of the B‐cell nature of HRS cells [57]. Based on these findings, the previous designation *Hodgkin's disease* was changed to Hodgkin lymphoma (cHL) [18].

## 5 CD30 Expression in Normal and Pathological Tissues

### 5.1 CD30 Expression in Normal Tissues

In normal lymphoid tissues, CD30 is expressed in a small subset of large mononuclear cells with evident nucleoli [13]. These large CD30‐positive cells are in the cell cycle as evidenced by Ki‐67 expression [58] and are mostly localized around B‐cell follicles and, to a minor degree, at the edge of germinal centers of tonsil, lymph nodes, and spleen, and around the Hassal's corpuscles of thymus [13, 16, 19, 21]. The cytological features of CD30+ cells and their topographical distribution in normal lymphoid tissues recall that of HRS cells in cHL. Therefore, it has been suggested that these elements might represent the normal counterpart of the neoplastic population of cHL [13, 16, 21]. Immunophenotypic and *IgV* gene analyses demonstrated that the rare germinal center and extrafollicular CD30+ cells represent B cells [59]. The transcriptomes of CD30+ germinal and extrafollicular B cells shared a strong MYC signature that differed from those of the CD30‐negative GC B cells, memory B, and plasma cells. CD30+ germinal center B cells may represent MYC+ centrocytes redifferentiating into centroblasts, whilst CD30+ extrafollicular B cells may consist of active, proliferating memory B cells [59]. By further investigations, it was found that the many CD30‐positive cells in reactive lymph nodes are a heterogenous population of polyclonal B cells, leading to the conclusion that there is no indication that such CD30‐positive B‐cell populations represent precursor lesions of Hodgkin lymphoma [60].

## 6 The Diagnostic Impact of CD30 Molecule

**FIGURE 4:**
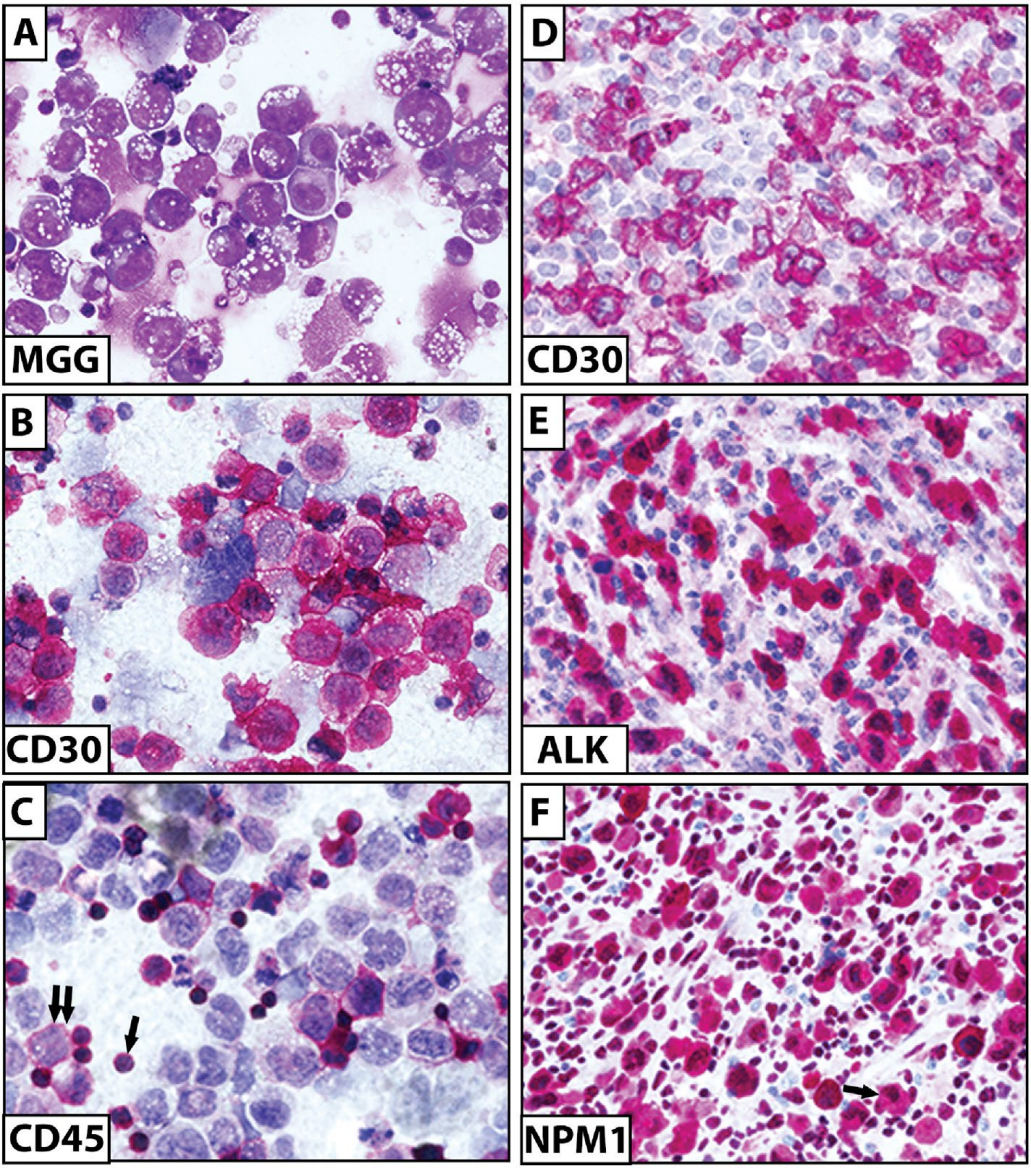
Differential diagnosis of ALCL. (A) Anaplastic large cell lymphoma ALK‐negative (ascitic fluid). Numerous large neoplastic cells with basophilic cytoplasm and cytoplasmic vacuoles are seen (May‐Grunwald‐Giemsa; ×400); (B) Most tumor cells are CD30‐positive (APAAP technique; ×400); (C) CD45 is expressed in small reactive lymphocytes (single arrow) but in only rare tumor cells (double arrows) (APAAP technique; ×400); (D) Anaplastic large cell lymphoma ALK‐positive. Immunostaining for CD30 (APAAP technique; ×400); (E) Immunostaining for ALK showing cytoplasmic and nuclear positivity of large tumor cells (APAAP technique; ×400); (F) Immunostaining for NPM1 showing cytoplasmic and nuclear positivity of large tumor cells (single arrow) and numerous normal reactive cells with nucleus‐restricted reactivity (APAAP technique; ×400). [Color figure can be viewed at wileyonlinelibrary.com]

CD30 expression has been detected in various lymphoid malignancies, the highest and most consistent expression being observed in cHL and ALCL. So the detection of high levels of CD30 is a must for the diagnosis of cHL and ALCL (Figure 4). More recently, the role of flow cytometry detection of CD30 for the diagnosis of breast implant‐associated ALCL has been assessed [61]. It is important to note that the presence of CD30‐positive cells in peri‐implant fluid does not equate to a diagnosis of lymphoma unless atypical cells are present. Variable expression and intensity of CD30 have been found in T‐cell lymphomas, like PTCL, cutaneous T‐cell lymphoma (CTCL), and extranodal NK‐T‐cell lym [62, 63, 64, 65, 66, 67, 68, 69, 70, 71, 72].

CD30 expression can also be a hint for the presence of EBV‐infected cells in malignant lymphoproliferations like EBV‐positive mucocutaneous ulcer and EBV‐positive DLBCL, as well as in non‐malignant lymphoproliferations like infectious mononucleosis [71] and chronic EBV‐driven lesions. Frequent CD30 expression also occurs in aggressive systemic mast cell tumors and leukemia, soft tissue tumors with kinase gene fusions, and germ cell tumors, most consistently in embryonal carcinoma [73, 74, 75, 76, 77]. Table 1 shows the lymphoma entities, other tumors, and non‐neoplastic lesions in which CD30 expression has strong, moderate, or only little or no diagnostic importance.

**TABLE 1:** Diagnostic importance of CD30 expression in lymphomas and non‐lymphoid disorders.

Lymphoma entities/lesions	% of cases	% of CD30 positivity
Strong diagnostic importance of CD30		
Classic Hodgkin Lymphoma (cHL)^a^	100%	100% +
Nodular sclerosis cHL	100%	100% +
Lymphocyte‐rich cHL	100%	100% +
Mixed‐cellularity cHL	100%	100% +
Lymphocyted depleted cHL	100%	100% +
Anaplastic Large Cell Lymphoma (ALCL)^a^		
Systemic ALCL	100%	100% +
ALK‐positive ALCL		
Large cell variant	100%	100% +
Small cell variant	100%	Range 50 to 5% +^b^
Breast implant‐associated ALCL	100%	100% +
Lymphomatoid papulosis	100%	100%+
Primary cutaneous ALCL	100%	100% +
Primary mediastinal (thymic) large B‐cell lymphoma	100%	> 80% +
Mediastinal gray‐zone lymphoma/B‐cell lymphoma	100%	> 95% +
Moderate diagnostic importance of CD30		
Nodal T‐ helper (TFH) cell lymphoma	> 50%	> 50% of large cells+
Angioimmunoblastic lymhoma	100%	> 70% of large cells+
Peripheral T‐cell lymphoma, NOS	25%	Only larger cells+
Adult T‐cell leukemia/lymphoma	100%	Cells variably+
Extranodal NK/T‐cell lymphoma	100%	> 50% +
Advanced stage mycosis fungoides	100%	Partially+
Primary cutaneous T‐cell lymphoma	30%	Only large cells+
Enteropathy‐associated T‐cell lymphoma	> 50%?	Large cells+
Intestinal T‐cell lymphoma, NOS	> 50%?	Large cells+
ALK‐positive diffuse large B‐cell lymphoma	< 15%	Cells variably+
Lymphomatoid granulomatosis, grade 3	> 90%	Large cells variably+
EBV+ diffuse large B‐cell lymphoma	> 90%	Large cells+
Plasmablastic lymphoma	30%	Most cells+
Fluid overload‐associated large B‐cell lymphoma	30%	Cells variably+
Lymphoid proliferations/lymphomas associated with immune deficiency and dysregulation	100%	HRS‐like cells+
EBV‐positive mucocutaneous ulcer	100%	Most cells+
Low or no diagnostic importance of CD30		
Diffuse large B‐cell lymphoma associated with		
Chronic inflamation	No data	Some cells+
Fibrin	No data	Some cells+
Fluid overload	No data	Some cells+
EBV‐positive conditions		
Infectious mononucleosis	Most cases	Many cells
Chronic active EBV‐infection (CAEBV)	Most cases	Many cells
Non‐lymphoid tumors		
Systemic mastocytosis high grade and mast cell leukemia	Most cases	Most cells+
Soft tissue tumors with kinase gene fusions, e.g., NTRK, BRAF, RAF1, RET	Most cases	Cells variably+
Embryonal carcinoma	> 70%	Most cells+

^a^ Strong CD30 expression in the majority of tumor cells is required for diagnosis of cHL and ALCL.

^b^ Usually the large tumor cells surround the vessels.

## 7 Development of Anti‐CD30 Immunotoxin

Because of its strong and consistent expression in HRS and ALCL cells and the limited expression in normal lymphoid tissues, the CD30 molecule was considered a suitable immunotherapeutic target [21, 35, 78].

### 7.1 Development of First Anti‐CD30 Immunotoxins (1992)

Pre‐clinical studies with the native anti‐CD30 mAbs SGN‐30 and 5F11 had shown promising activity in vitro and in mouse models [79, 80, 81], but only demonstrated scarce anti‐tumor efficacy in patients [82, 83, 84].

We investigated the activity of anti‐CD30 antibodies conjugated with the plant toxin saporin or other toxins preclinically [78, 85, 86] and demonstrated for the first time that this immunotoxin was active in patients with refractory/relapsed cHL [87]. However, relapses occurred frequently because repeated treatments were not possible due to the strong host immune response both against saporin and ricin and the murine antibody moieties [87, 88].

### 7.2 Development of a Potent Anti‐CD30 Monoclonal Antibody Auristatin Conjugate (2003)

Eleven years later, the non‐immunogenic toxin monomethyl auristatin E (MMAE) was linked to a humanized anti‐CD30 antibody. Auristatin is a microtubule inhibitor [89, 90]. The anti‐CD30 auristatin conjugate binds to the surface CD30 of neoplastic cells and is internalized through receptor endocytosis and captured into lysosomes where the auristatin is released by proteolytic enzymes and becomes toxic. The released auristatin inhibits the polymerization of tubulin in the cellular cytoskeleton, with consequent cell cycle arrest in G2/M and apoptosis or antibody‐dependent cellular phagocytosis. Auristatin can also induce the death of neighboring cells by diffusion across the cell membrane [91, 92]. Moreover, auristatin seems to exert additional anti‐tumor immunity by stimulation of dendritic cells [93]. The conjugate was named brentuximab vedotin (BV) and has shown significant in vitro and in vivo activity in preclinical and clinical studies [89, 94, 95].

Despite its high specificity, BV has off‐target effects, especially in terms of peripheral neuropathy that is likely mediated through the anti‐tubulin effects of auristatin [91]. Other, more rare, but serious side effects include pancreatitis and leukencephalopathy. Various resistance mechanisms to BV have been reported in cHL and ALCL cell lines, including increased expression of drug transporter proteins [96], auristatin E resistance [97] and release by HRS cells of CD30‐containing extracellular vesicles [98]. CD30 downregulation has been observed in vitro but not in tissue samples of cHL [99]. However, downregulation of CD30 following BV has been reported in refractory (relapsed (R/R)) ALCL cases [100, 101, 102].

## 8 CD30 as a Target in cHL

Outcomes for patients with advanced‐stage cHL have improved dramatically over the past half century. However, up to 30% of patients with stage III–IV cHL show refractory disease or relapse after frontline chemotherapy, and the outcome is even worse in older patients. During the last two decades, further progress has been made using anti‐CD30 immunotherapy.

### 8.1 Brentuximab Vedotin Plus Chemotherapy Reshapes Frontline Treatment of Advanced Stage cHL

Until recently, ABVD (doxorubicin, bleomycin, vinblastine, and dacarbazine) was the standard of care in cHL in the USA. The German Hodgkin Study Group (GHSG) used mainly escalated BEACOPP (bleomycin, etoposide, doxorubicin, cyclophosphamide, vincristine, procarbazine, and prednisone) (esc‐B) for advanced cHL.

A change in the standard of care in this disease was the inclusion of BV in the first‐line treatment [94, 103]. The ECHELON‐1 phase 3 trial compared in stage III–IV cHL, BV‐AVD (*n* = 664 patients) to ABVD (*n* = 670 patients) [94, 104]. The 2‐year modified PFS was 82.1% for BV‐AVD and 77.2% for ABVD (*p* = 0.04) [104]. There was a higher incidence of neutropenia (58% versus 45%) and ≥ 2 degree peripheral neuropathy (31% versus 11%) in BV‐AVD vs. ABVD. Pulmonary toxicity of grade 3 or higher was reported in < 1% of patients receiving BV‐AVD and in 3% of those receiving ABVD. At 6‐year follow‐up, BV‐AVD had a 7.8% PFS and a 4.5% OS benefit over ABVD [105, 106].

BrECADD (BV, etoposide, doxorubicin, cyclophosphamide, dacarbazine, dexamethasone) was compared with escalated BEACOPP (esc‐B) in the PET‐adapted phase 3 HD21 trial [107, 108]. Sixty‐four percent of patients were iPET negative and received a total of 4 cycles of therapy. As compared to esc‐B, BrECADD showed a superior 4‐year PFS (94.3% versus 90.9%), lower acute hematologic toxicity (G4 31% versus 52%), and lower peripheral sensory neuropathy (all grades 38% versus 49%). There was no significant residual organ toxicity at 1 year after treatment, and > 95% of female patients had hormonal recovery [109]. Based on these results, BrECADD is the new standard of care within the GHSG for adult cHL patients with advanced stage disease, age ≤ 60 years.

Older patients with advanced cHL, especially those unfit for chemotherapy, usually have worse outcomes than younger patients [110]. However, BV monotherapy [111, 112] or sequential BV‐AVD [113] has shown promising results in this setting. With sequential BV‐AVD, the 2‐year PFS and OS were 84% and 93%, respectively. Patients with cHL > 60 years unfit for chemotherapy may also benefit from BV given in combination with dacarbazine or nivolumab [114]. The response rates were high, and, with a median follow‐up of > 4 years, nearly one‐half of responses were durable [114]. Long‐term follow‐up with BV plus nivolumab indicates that this regimen is effective for patients > 60 years, with a cure rate of approximately 40% [115]. BV‐AVD was highly active and had a tolerable adverse event rate even in HIV‐related, advanced‐stage cHL [116, 117].

### 8.2 Reshaping Frontline Treatment of Early‐Stage cHL

BV combinations were evaluated in early‐stage unfavorable cHL with the aim of increasing the CR rates, reducing the duration of therapy, or omitting radiation therapy. In the BREACH study [118], four courses of BV‐AVD followed by involved nodal radiotherapy (30 Gy) were associated with higher PET‐2 negative rates as compared to 4 ABVD + 30 Gy (82.3% vs. 75.4%). However, 2‐year PFS was similar (97.3% versus 92.6%). Febrile neutropenia and peripheral neuropathy were more frequent with BV‐AVD. In another study, patients with newly diagnosed, early‐stage cHL with unfavorable risk were treated with 4 cycles of BV‐AVD, and those achieving negative PET received 30 Gy in situ radiation therapy [119]. More than 90% of patients achieved negative interim PET after 2–4 cycles of BV‐AVD [119]. All 25 patients who completed BV + AVD + in situ radiation therapy achieved a CR. With a median follow‐up of 18.8 months, by intent to treat, the 1‐year PFS was 93.3%. The treatment was well‐tolerated with no significant pulmonary toxicity. This may be a highly active regimen, especially in patients with bulky disease. In a phase 2 study, ABVD (2–6 cycles) followed by consolidation BV (6 cycles) was evaluated in unfavorable risk, early‐stage cHL [120]. The CR rate was 95% with an estimated 3‐year PFS of 92%.

BV‐AVD was also evaluated for non‐bulky stage I/II cHL [121] resulting in a high CR rate and PFS and OS of 94% and 97%, respectively, with most patients requiring only 4 cycles of therapy. Because toxicity was higher than would be expected from AVD alone, this approach may not be appropriate for early‐stage patients with a favorable prognosis. Finally, a study evaluated the omission of bleomycin and vinblastine (BV‐DC) in non‐bulky limited‐stage cHL. A high CR rate and durable PFS were observed, with most patients requiring 4 cycles of therapy [122]. The estimated 5‐year PFS and OS were 91% and 96%, respectively.

Cumulatively, all the above studies suggest that high CR rates and excellent PFS may be obtained by incorporating BV, allowing a chemotherapy‐only treatment of early‐stage cHL.

### 8.3 Brentuximab Vedotin in Refractory/Relapsed (R/R) cHL

R/R cHL is usually treated with non–cross‐resistant chemotherapy regimens followed by autologous stem cell transplantation (ASCT) in chemosensitive patients. This combination usually results in about 50% long‐term cure rates. BV in R/R cHL was evaluated in two studies, one of phase 1 [123] and the other of phase 2 [124], both showing safety and activity. In the largest study of phase 2, 102 patients with R/R cHL after ASCT were enrolled [125]. The ORR was 75% with 34% CR. The median PFS for all patients was 5.6 months, and the median duration of response for those in CR was 20.5 months. After 1.5 years, 31 patients were alive and free of documented progressive disease. The most common treatment‐related adverse events were peripheral sensory neuropathy, nausea, fatigue, neutropenia, and diarrhea. Five‐year follow‐up data demonstrate that a subset of patients with R/R cHL who obtained CR with single‐agent brentuximab vedotin had long‐term disease control and may potentially be cured [124].

BV as a single, second‐line agent may be an effective bridge to ASCT, with an ORR of 75% and a CR rate of 43% [126]. After ASCT, the 2‐year PFS and OS were 67% and 93%, respectively. Pre‐transplantation PET negativity was one of the strongest predictors of outcome post‐transplant. A multicenter phase 2 study reported an ORR of 68% after BV x 4, and with this approach, 49% of patients (18/37) avoided additional chemotherapy, proceeding directly to ASCT after CR (35%) or partial response (14%) [127]. BV plus nivolumab was also safe and effective as a bridge to ASCT, with an ORR of 85% and a CR rate of 67%. OS at 3 years was 93% [128].

PET‐adapted sequential salvage therapy with BV followed by augmented ICE resulted in a high rate of PET‐negativity, with a 2‐year PFS of 80% post‐transplant [129]. Lynch et al. [130] observed similar outcomes combining dose‐dense BV and ICE. Due to its significant toxicity, this approach may be a second‐line therapy option in younger transplantation‐eligible patients. In addition to ICE, BV was combined with other chemotherapy regimens, resulting in CR rates of 70%–79% and 2‐year PFS rates of 70%–76% after ASCT [131, 132, 133]. However, these regimens are associated with a high rate of grade 3–4 hematological toxicity. BV with or without salvage chemotherapy appears to enhance PFS in cHL patients with relapsed disease but not in those who are primary refractory [134].

BV as consolidation after ASCT represents a treatment option for cHL patients with unfavorable risk factors (refractory disease, relapse < 1 year after initial treatment, extranodal disease at relapse) who usually progress after ASCT [135]. The randomized, double‐blind, phase 3 AETHERA trial evaluated the safety and efficacy of BV as consolidation in this setting [136]. In this study, 329 patients with R/R unfavorable‐risk cHL were randomly assigned to receive either BV or placebo for up to 16 cycles after ASCT. The median PFS in the BV and placebo groups was 42.9 and 24.1 months, respectively [136]. However, no significant difference in OS was observed between BV and placebo arms [136]. At a further follow‐up, BV continued to provide patients with sustained PFS benefit, 5‐year PFS being 59% with BV vs. 41% with placebo [137]. These results led to the FDA approval of BV as post‐ASCT consolidation in cHL patients at high risk of relapse or progression.

Cumulatively, the above studies suggest that BV can be combined safely with chemotherapy, resulting in high CR rates and a potential to improve outcomes with ASCT [105].

## 9 Targeted Therapy of CD30+ Peripheral T‐Cell Lymphomas

### 9.1 Reshaping Frontline Treatment of CD30+ ALCL and Other PTCL

CHOP represents the standard front‐line therapy for patients with PTCL, but this regimen is associated with frequent relapses and a 5‐year survival of only 35% [138, 139]. To improve clinical results, BV has been combined with chemotherapy in the frontline setting [140, 141, 142]. The phase III ECHELON‐2 compared BV + cyclophosphamide, doxorubicin, and prednisone (CHP) vs. CHOP in patients with ALCL or other CD30‐positive PTCL with > 10% CD30 expression [143]. At the 5‐year follow‐up, BV‐CHP was superior to CHOP, with improved PFS (51.4% vs. 43%) and OS (70.1% vs. 61%) [144]. A further analysis of ECHELON‐2 showed that improved outcomes at 5 years of follow‐up were observed with A + CHP vs. CHOP in both ALK+ and ALK−subgroups [145].

BV‐CHP is clearly the standard of care option for ALCL that show strong expression for CD30 whilst the benefit of this regimen in PTCL‐NOS and AITL subgroups that express CD30 not uniformly is uncertain. The impact of consolidative ASCT after BV + CHP in patients achieving CR at the end of treatment was assessed in a subgroup analysis of the ECHELON‐2 trial, which reported better PFS with ASCT than without (5‐year PFS 65.3% vs. 46.4%) [144]. These results were also confirmed in another study [146].

CHOEP has higher activity than CHOP, especially in ALK+ ALCL [139]. The safety and efficacy of CHEP‐BV followed by BV consolidation have been evaluated in 46 patients with CD30‐expressing PTCL. ORR was 91% and CR was 80%, regardless of subtype or CD30 expression [147]. This regimen may have a role in young, high‐risk patients. These data were confirmed in a more recent study [148]. CHEP‐BV was safe and effective even as frontline therapy in adult T cell lymphoma/leukemia, when used as a bridge to allogeneic hematopoietic stem cell transplantation [149].

BV in CD30+ cutaneous T‐cell lymphoma and lymphomatoid papulosis is well tolerated and results in an ORR of 73% and a CR of 35% [150]. The activity and safety of BV versus physician's choice of either methotrexate or bexarotene were evaluated in cutaneous T cell lymphomas in a phase III trial [151]. Significant improvement in objective response lasting at least 4 months was seen with BV vs. physician's choice of methotrexate or bexarotene (56.3% vs. 12.5%) [151].

### 9.2 BV in R/R CD30+ ALCL and Other PTCL

About half of CD30+ ALCL patients, especially the ALK‐negative form and an even higher percentage of other CD30+ PTCL subtypes, relapse after front‐line treatment and consolidation with ASCT [152, 153, 154]. Therefore, BV has been widely used in the R/R setting. The safety and efficacy of BV as monotherapy (1.8 mg/kg, once every 3 weeks for up to 16 cycles) were evaluated in a Phase II study in 58 patients (median age 52 years) with R/R ALCL, mostly ALK‐negative [155]. The ORR was 86% in patients who had failed prior therapies including ASCT, with 57% CR. The median duration of the response was 13.2 months for CR. Neutropenia, thrombocytopenia, and peripheral sensory neuropathy were the principal grade 3–4 adverse events affecting > 10% of patients. At a 5‐year follow‐up, 38 (66%) patients were in CR, independently of ALK status or number of prior therapies [156]. The estimated OS rate for the whole series and the subset of patients not achieving CR was 79% and 25%, respectively [156]. Moreover, the PFS rate among CR patients was substantially higher than in total enrolled patients (57% versus 39%, respectively). Similar results with an ORR of 62.5% (45% CR and 17.5% PR) were reported by an Italian observational, multicenter, retrospective study in 40 patients with systemic ALCL [157].

Activity of BV monotherapy in other types of R/R PTCL is inferior as compared to ALCL, with ORR being more frequently observed in 54% of AITL cases [140, 158]. The median duration of response was limited. BV has been also used in combination with bendamustine in R/R PTCL patients (*n* = 82) [159]. The best ORR was 68%, with 49% CR. Median duration of response was 15.4 months for patients in CR. The combination BV + ICE (ifosfamide, carboplatin, etoposide) in R/R PTCL was associated with an ORR of 66.7%, with all patients achieving a CR [160]. The activity of BV plus nivolumab in R/R PTCL and cutaneous T‐cell lymphoma has been more modest [161].

## 10 Other CD30 Expressing Lymphomas

CD30 is variably expressed in DLBCL and mediastinal lymphomas, and the activity and safety of BV have been documented in R/R DLBCL, both as monotherapy [162] with 44% ORR (including 17% CR) or in combination with lenalidomide, with 57% ORR and 35% CR [163]. The combination of BV + nivolumab was studied in R/R primary mediastinal B‐cell lymphomas. After a median follow‐up of 11.1 months, the ORR was 73%, with a CR rate of 37% [164]. Due to the high success of anti‐CD19 chimeric antigen receptor (CAR) T cells in R/R DLBCL and primary mediastinal lymphomas, there has been a decreased interest in BV in this setting. However, this agent may still have a role as bridge therapy to CAR‐T cells.

## 11 Anti‐CD30 CAR‐T Cells

### 11.1 Treatment of R/R cHL and Other CD30+ Lymphomas

CAR‐T cells directed against CD19 have revolutionized the treatment of R/R B‐cell acute lymphoblastic leukemia and B‐cell lymphomas [165, 166]. Results of CD30‐directed CAR‐T cells in R/R cHL have been more modest [167]. In the first published phase I trial [168], 18 heavily pretreated patients (17 with R/R cHL and 1 with ALCL) received anti‐CD30 CAR‐T cells. The ORR was 39% (all partial responders) and the median PFS was 6 months. All patients experienced grade 1–2 cytokine release syndrome (CRS) but not immune effector cell associated neurotoxicity syndrome (ICANS) or treatment‐related deaths. Other studies showed similar unsatisfactory responses [169, 170], associated with high toxicity [170].

In a phase I/II trial [171], 41 patients with heavily pre‐treated cHL received anti‐CD30 CAR‐T cells. The ORR of the whole cohort was 62% with a 1‐year PFS of 36%. Among the 32 patients who received a fludarabine‐based lymphodepletion, ORR was 72%, with 19 patients (59%) achieving CR; the 1‐year PFS and OS of this subgroup were 61% and 94%, respectively. CRS was of low grade, and no neurologic toxicity was observed. These favorable results were confirmed in 15 refractory cHL patients with a CR rate of about 60% [172, 173]. However, a high CR rate in cHL is usually associated, at a follow‐up of 6 years, with a low PFS of only 19% and a duration of response of 25% [174].

ASCT was used in tandem with anti‐CD30 CAR‐T cell infusion to treat R/R CD30+ lymphomas [175]. This approach is well‐tolerated and highly effective in R/R cHL and ALCL, even in PET‐positive or chemorefractory patients who are expected to have inferior outcomes after ASCT.

Third‐generation anti‐CD30 CAR‐T cells were evaluated in combination with lymphodepletion in R/R CD30^+^ lymphoma [176]. The median PFS for the 9 patients was 13 months, with three long‐term CRs over 2 years response. This may be explained by the two costimulatory domains CD28 and 4‐1BB [176] that can both facilitate CAR‐T cell proliferation (due to the CD28 costimulatory domain) and prolong CAR‐T cell persistence in vivo (due to the 4‐1BB endodomain) [177, 178]. In another study [179] CD30.CAR‐Ts co‐expressing the cognate receptor for CCL17, CCR4 (CCR4.CD30.CAR‐Ts) with improved tumor homing and anti‐lymphoma activity compared with CD30.CAR‐Ts not expressing CCR4 were used to treat 8 R/R cHL patients, with 6 (75%) achieving a CR and 2 (25%) a PR. Five patients are in remission to date, with one patient still in CR at 2.5 years post treatment.

Anti‐CD30 CAR T cells have also been used as consolidation after ASCT in CD30‐positive lymphoma patients at high risk of relapse [180]. In particular, 18 patients (11 cHL, six T‐cell lymphoma, one gray zone lymphoma) were infused with anti‐CD30 CAR‐T cells at a median of 22 days (range 16–44) after ASCT. One patient had grade 1 CRS. The most common grade 3–4 adverse events were lymphopenia and leukopenia (11% of cases). At a median follow‐up of 48.2 months post‐infusion, the median PFS for all treated patients (*n* = 18) was 32.3 months and the median PFS for treated patients with cHL (*n* = 11) has not been reached [180]. The median OS for all treated patients has not been reached.

Despite the high response rates to anti‐CD30 CAR‐T cells, when preceded by lymphodepleting chemotherapy, disease progression is common, with treatment failures being mainly correlated to higher metabolic tumor volume (> 60 mL) by PET before CAR‐T cell infusion [181]. Conversely, bridging therapy, anti‐CD30 CAR‐T cell expansion/persistence, and the percentage of CD3+ lymphocytes over the first 6 weeks of therapy did not impact PFS.

A recent meta‐analysis on the safety and efficacy of anti‐CD30 CAR‐T cell therapy in R/R cHL was performed on a total of 151 participants [182]. The ORR and CR were 57% and 34%, respectively. A partial response was observed in 32% of cases. With the median follow‐up range from 9.5 to 71.5 months, the 1‐year PFS was 39%, and the 1‐year OS was 89%. Leukopenia and CRS were the most common adverse events, but they were tolerable and resolved with treatment. Clinical trials with anti‐CD30 CAR‐T cells in cHL are summarized in Table 2.

**TABLE 2:** Clinical trials of CAR‐T cell therapy for classic Hodgkin lymphoma.

Phase	Patients no.	LD	Dose	Outcomes	CRS/ICANS	References
I	17	Fluda + Cyclo, Gem + must + Cyclo Nab‐paclitaxel + Cyclo	1 – 3 × 10^7^ CAR‐T cells/kg (median 1.56 × 10^7^)	ORR 70%, PR 35%, SD 35%	≥ 3 none	Wang et al. [168]
I	6	Fluda + Cyclo	0.7–3.2 × 10^7^ CAR‐T cells/kg (median 1.4 × 10^7^)	CR 83%	CRS ≥ 3 0% (1 grade 5 pleural hemorrhage) ICANS 0%	Wang et al. [176]
I	7	No LD	2 × 10^7^–2 × 10^8^ CAR‐T cells/kg	SD 43% CR 29% CR 50% 2 × 10^8^ per kg of CAR‐T cells	0%	Ramos et al. [169]
I/II	41	Benda monotherapy, Benda + Fluda, Fluda + Cyclo	2 × 10^7^, 1 × 10^8^, 2 × 10^8^ CAR‐T cells/m^2^	ORR 62% CR 51% (Benda + Fluda CR 73%) PR 11% SD 11%	CRS grade 1 34% ICANS 0%	Ramos et al. [171]
I/II	15	Fluda + Benda	2.0–2.7 × 10^8^ CAR‐T cells/m^2^	SI group: ORR 73% CR 60% RI group: ORR 100% CR 60%	CRS grade I 6% ICANS 0%	Ahmed et al. [172]
I	9	Fluda + Benda	3 × 10^6^, 5 × 10^6^, 10 × 10^6^ CAR‐T cells/kg	ORR 100% CR 62.5% 6 months PFS 75%	CRS grade I 60% ICANS 0%	Caballero‐Gonzalez et al. [173]
I/II^a^	11	Fluda + Benda	2 × 10^8^ CAR‐T/m^2^	2 years PFS 73%	1 patient CRS grade 1 ICANS 0%	Grover et al. [180]
I/II	4	Cyclo	< 80 kg received 8 × 10^6^ to 1.5 × 10^8^ CAR‐T cells/kg, ≥ 80 kg 1 × 10^8^ CAR‐T cells/kg	ORR 50% CR 25% PR 25% SD 25%	0%	Mei et al. [183]
I	5	BEAM ± Fluda	3.9 × 10^6^/kg 7.6 × 10^6^/kg	CR 75%	CRS grade 1100% ICANS 0%	Brudno et al. [170]
I^b^	20	Fluda + Cyclo	0.3 × 10^6^/kg 1 × 10^6^/kg 3 × 10^6^/kg 9 × 10^6^ /kg	ORR 43% CR 5%	CRS 52% ICANS 24%	Zhang et al. [170]
I	29	Benda, Fluda + benda	1 × 10^8^/m^2^ 2 × 10^8^ /m^2^	CR 79%	N/A	Reed et al. [174]

Abbreviations: Bendamustine = Benda; CR = complete response; CRS = cytokine releasing syndrome; Cyclophosphamide = Cyclo; Fludarabine = Fluda; Gemcitabine = Gem; Mustargen = Must.; ICANS = immune effector associated neurotoxicity syndrome; LD = lymphodepletion; N/A = not available; ORR = overall response rate; PFS = progression free survival; PR = partial response; RI = repeated infusion; SD = disease stabilization; SI = single infusion.

^a^ Anti‐CD30 CAR‐T cells as consolidation after autologous stem cell transplantation.

^b^ Autologous stem cell transplantation in tandem with anti‐CD30 CAR‐T cells infusion.

### 11.2 Mechanism of Resistance to CD30‐Directed CAR‐T Cells and Treatment of Relapses After CAR‐T Cells

Mechanisms of resistance to anti‐CD30 CAR‐T cells have been investigated. Decreased CD30 expression after BV and anti‐CD30 CAR‐T cells was observed in a patient with cHL [184]. Similar findings were reported by Marques‐Piubelli [185] but these were not confirmed [186]. Conversely, other investigators found that CD30 expression is usually retained in relapsing tumors after anti‐CD30 CAR‐T cell therapy. These findings suggest that the recurrence may be more likely due to the low affinity binding scFv to CD30 and/or to the highly immunosuppressive tumor microenvironment in cHL. Thus, strategies have been adopted to overcome these problems. Artificial intelligence and surface plasmon resonance were used to select scFv fragments of mAbs with high affinity for CD30 [187]. Dual anti‐CD30/PDL1‐CCR CAR‐T cells can be adopted as a strategy. In this way, PDL1.CCR is expected to block PDL1/PD1 interaction of CAR‐T cells and macrophages with PDL1+ tumor cells, mimicking an immune checkpoint effect and reducing CAR‐T cell exhaustion [188]. At the same time, stimulation of PDL1‐4.1 BB costimulatory receptor would enhance CAR‐T cell proliferation and persistence [188].

In patients relapsing after CAR‐T cell treatment, PD‐1 inhibitors may re‐induce CR, even in patients previously exposed to or progressed under these drugs [176]. In addition, anti‐CD30 CAR‐T cells can be combined with anti‐PD1 antibody therapy [167, 183, 189]. In particular, the anti‐PD‐1 antibody enhanced the effect of CD30‐directed CAR‐T therapy in R/R CD30+ lymphoma patients, with minimal toxicities [189]. The ORR was 91.7% (11/12), with 6 patients achieving CR (50%), and CRS was of low grade. With a median follow‐up of 21.5 months (range: 3_−_50 months), the PFS and the OS were 45% and 70%, respectively.

## 12 Conclusions

The CD30 molecule is an excellent diagnostic marker for cHL and ALCL. Targeting CD30 is also valuable for lymphoma treatment. The high CR rates observed when incorporating BV in the frontline treatment of early‐stage cHL suggest that this strategy may potentially lead to omitting radiotherapy in this setting. Patients with advanced stage cHL who are not eligible for anthracycline‐based chemotherapy may benefit from BV‐nivolumab. The activity of BV as frontline therapy in some PTCL categories needs to be further investigated, especially given the variability of CD30 expression across subtypes, with the highest levels being found in ALCL and cHL. The optimal CD30 expression cutoff for patient selection remains an unsolved issue [140, 190]. Responses to BV have been observed with low or even absent expression of CD30, as in various subtypes of T‐cell lymphomas and mycosis fungoides [150, 191, 192]. Such responses could be due to the presence of low levels of surface CD30, below the threshold of detection by immunohistochemistry. A more appealing hypothesis is the bystander effect of BV, where MMAE crosses the cell membrane of the rare killed CD30+ tumor cells and is released into the surrounding extracellular matrix, exerting its cytotoxic activity on adjacent CD30‐negative tumor cells [193]. The activity of anti‐CD30 CAR‐T cells has been so far modest, and attempts should be made to modify them structurally, with the aim to improve the anti‐tumor activity and also to antagonize the immunosuppressive microenvironment of cHL.

## Ethics Statement

The authors have nothing to report.

## Conflicts of Interest

The authors declare no conflicts of interest.

## Data Availability

The data that support the findings of this study are available from the corresponding author upon reasonable request.

## References

[1] K. Lennert , Pathologie der Halslymphknoten. Ein Abriss für Pathologen, Kliniker and praktizierende Ärztre (Springer, 1964).

[2] H. Rappaport , Tumors of the Hematopoietic System. Atlas of Tumor Pathology (Armed Forced Institute of Pathology, 1966).

[3] M. D. Cooper , R. D. Peterson , and R. A. Good , “Delineation of the Thymic and Bursal Lymphoid Systems in the Chicken,” Nature 205 (1965): 143–146. 14276257 10.1038/205143a0

[4] M. C. Raff , M. Sternberg , and R. B. Taylor , “Immunoglobulin Determinants on the Surface of Mouse Lymphoid Cells,” Nature 225, no. 5232 (1970): 553–554. 4189355 10.1038/225553a0

[5] H. Stein , K. Lennert , and M. R. Parwaresch , “Malignant Lymphomas of B‐Cell Type,” Lancet 2, no. 7782 (1972): 855–857. 4116558 10.1016/s0140-6736(72)92215-5

[6] H. Stein , E. Kaiserling , and K. Lennert , “Evidence for B‐Cell Origin of Reticulum Cell Sarcoma,” Virchows Archiv. A, Pathological Anatomy and Histology 364, no. 1 (1974): 51–67. 4374798 10.1007/BF01230857

[7] H. S. Kaplan and S. Gartner , “Sternberg‐Reed Giant Cells of Hodgkin's Disease: Cultivation In Vitro, Heterotransplantation, and Characterization as Neoplastic Macrophages,” International Journal of Cancer 19, no. 4 (1977): 511–525. 844918 10.1002/ijc.2910190412

[8] J. C. Long , P. C. Zamecnik , A. C. Aisenberg , and L. Atkins , “Tissue Culture Studies in Hodgkin's Disease: Morphologic, Cytogenetic, Cell Surface, and Enzymatic Properties of Cultures Derived From Splenic Tumors,” Journal of Experimental Medicine 145, no. 6 (1977): 1484–1500. 68093 10.1084/jem.145.6.1484 PMC2180683

[9] J. C. Long , C. L. Hall , C. A. Brown , C. Stamatos , S. A. Weitzman , and K. Carey , “Binding of Soluble Immune Complexes in Serum of Patients With Hodgkin's Disease to Tissue Cultures Derived From the Tumor,” New England Journal of Medicine 297, no. 6 (1977): 295–299. 559938 10.1056/NEJM197708112970602

[10] N. L. Harris , D. L. Gang , S. C. Quay , et al., “Contamination of Hodgkin's Disease Cell Cultures,” Nature 289, no. 5795 (1981): 228–230. 7192801 10.1038/289228a0

[11] M. Schaadt , C. Fonatsch , H. Kirchner , and V. Diehl , “Establishment of a Malignant, Epstein‐Barr‐Virus (EBV)‐Negative Cell‐Line From the Pleura Effusion of a Patient With Hodgkin's Disease,” Blut 38, no. 2 (1979): 185–190. 216443 10.1007/BF01007965

[12] H. Stein , J. Gerdes , H. Kirchner , M. Schaadt , and V. Diehl , “Hodgkin and Sternberg‐Reed Cell Antigen(s) Detected by an Antiserum to a Cell Line (L428) Derived From Hodgkin's Disease,” International Journal of Cancer 28, no. 4 (1981): 425–429. 6946981 10.1002/ijc.2910280406

[13] U. Schwab , H. Stein , J. Gerdes , et al., “Production of a Monoclonal Antibody Specific for Hodgkin and Sternberg‐Reed Cells of Hodgkin's Disease and a Subset of Normal Lymphoid Cells,” Nature 299, no. 5878 (1982): 65–67. 7110326 10.1038/299065a0

[14] H. Stein , J. Gerdes , U. Schwab , et al., “Identification of Hodgkin and Sternberg‐Reed Cells as a Unique Cell Type Derived From a Newly‐Detected Small‐Cell Population,” International Journal of Cancer 30, no. 4 (1982): 445–459. 6754630 10.1002/ijc.2910300411

[15] A. Bernard , P. C. L. Beverley , L. Boumsell , et al., “Interim Report of the Third Workshop,” in Leucocyte Typing III, White Cell Differentiation Antigens (Oxford University Press, 1987).

[16] R. Schwarting , J. Gerdes , H. Durkop , B. Falini , S. Pileri , and H. Stein , “BER‐H2: A New Anti‐Ki‐1 (CD30) Monoclonal Antibody Directed at a Formol‐Resistant Epitope,” Blood 74, no. 5 (1989): 1678–1689. 2477085

[17] N. L. Harris , E. S. Jaffe , H. Stein , et al., “A Revised European‐American Classification of Lymphoid Neoplasms: A Proposal From the International Lymphoma Study Group,” Blood 84, no. 5 (1994): 1361–1392. 8068936

[18] E. Jaffe , N. L. Harris , H. Stein , and J. W. Vardiman , Tumours of Haematopoietic and Lymphoid Tissue (IARC press, 2001).

[19] H. Stein , D. Y. Mason , J. Gerdes , et al., “The Expression of the Hodgkin's Disease Associated Antigen Ki‐1 in Reactive and Neoplastic Lymphoid Tissue: Evidence That Reed‐Sternberg Cells and Histiocytic Malignancies Are Derived From Activated Lymphoid Cells,” Blood 66, no. 4 (1985): 848–858. 3876124

[20] J. Rodriguez , W. Pugh , J. Romaguera , and F. Cabanillas , “Anaplastic Ki‐1 + Large‐Cell Lymphoma,” Cancer Investigation 11, no. 5 (1993): 554–558. 8402224 10.3109/07357909309011673

[21] B. Falini , S. Pileri , G. Pizzolo , et al., “CD30 (Ki‐1) Molecule: A New Cytokine Receptor of the Tumor Necrosis Factor Receptor Superfamily as a Tool for Diagnosis and Immunotherapy,” Blood 85, no. 1 (1995): 1–14. 7803786

[22] H. D. Foss , I. Anagnostopoulos , I. Araujo , et al., “Anaplastic Large‐Cell Lymphomas of T‐Cell and Null‐Cell Phenotype Express Cytotoxic Molecules,” Blood 88, no. 10 (1996): 4005–4011. 8916967

[23] C. Fonatsch , U. Latza , H. Durkop , H. Rieder , and H. Stein , “Assignment of the Human CD30 (Ki‐1) Gene to 1p36,” Genomics 14, no. 3 (1992): 825–826. 1330892 10.1016/s0888-7543(05)80203-4

[24] O. Kemper , J. Derre , D. Cherif , H. Engelmann , D. Wallach , and R. Berger , “The Gene for the Type II (p75) Tumor Necrosis Factor Receptor (TNF‐RII) is Localized on Band 1p36.2‐p36.3,” Human Genetics 87, no. 5 (1991): 623–624. 1655619 10.1007/BF00209026

[25] H. Durkop , U. Latza , M. Hummel , F. Eitelbach , B. Seed , and H. Stein , “Molecular Cloning and Expression of a New Member of the Nerve Growth Factor Receptor Family That Is Characteristic for Hodgkin's Disease,” Cell 68, no. 3 (1992): 421–427. 1310894 10.1016/0092-8674(92)90180-k

[26] C. A. Smith , H. J. Gruss , T. Davis , et al., “CD30 Antigen, a Marker for Hodgkin's Lymphoma, Is a Receptor Whose Ligand Defines an Emerging Family of Cytokines With Homology to TNF,” Cell 73, no. 7 (1993): 1349–1360. 8391931 10.1016/0092-8674(93)90361-s

[27] L. Dong , M. Hulsmeyer , H. Durkop , et al., “Human CD30: Structural Implications From Epitope Mapping and Modeling Studies,” Journal of Molecular Recognition 16, no. 1 (2003): 28–36. 12557237 10.1002/jmr.605

[28] Y. Mukai , T. Nakamura , M. Yoshikawa , et al., “Solution of the Structure of the TNF‐TNFR2 Complex,” Science Signaling 3, no. 148 (2010): ra83. 21081755 10.1126/scisignal.2000954

[29] S. L. Buchan and A. Al‐Shamkhani , “Distinct Motifs in the Intracellular Domain of Human CD30 Differentially Activate Canonical and Alternative Transcription Factor NF‐kappaB Signaling,” PLoS One 7, no. 9 (2012): e45244. 23028875 10.1371/journal.pone.0045244 PMC3445475

[30] R. W. Gedrich , M. C. Gilfillan , C. S. Duckett , J. L. Van Dongen , and C. B. Thompson , “CD30 Contains Two Binding Sites With Different Specificities for Members of the Tumor Necrosis Factor Receptor‐Associated Factor Family of Signal Transducing Proteins,” Journal of Biological Chemistry 271, no. 22 (1996): 12852–12858. 8662842 10.1074/jbc.271.22.12852

[31] J. F. Nawrocki , E. S. Kirsten , and R. I. Fisher , “Biochemical and Structural Properties of a Hodgkin's Disease‐Related Membrane Protein,” Journal of Immunology 141, no. 2 (1988): 672–680. 3385212

[32] P. Froese , H. Lemke , J. Gerdes , et al., “Biochemical Characterization and Biosynthesis of the Ki‐1 Antigen in Hodgkin‐Derived and Virus‐Transformed Human B and T Lymphoid Cell Lines,” Journal of Immunology 139, no. 6 (1987): 2081–2087. 3040864

[33] G. Pizzolo , F. Vinante , M. Chilosi , et al., “Serum Levels of Soluble CD30 Molecule (Ki‐1 Antigen) in Hodgkin's Disease: Relationship With Disease Activity and Clinical Stage,” British Journal of Haematology 75, no. 2 (1990): 282–284. 2164839 10.1111/j.1365-2141.1990.tb02664.x

[34] G. Nadali , F. Vinante , H. Stein , et al., “Serum Levels of the Soluble Form of CD30 Molecule as a Tumor Marker in CD30+ Anaplastic Large‐Cell Lymphoma,” Journal of Clinical Oncology 13, no. 6 (1995): 1355–1360. 7751879 10.1200/JCO.1995.13.6.1355

[35] B. Falini , L. Flenghi , L. Fedeli , et al., “In Vivo Targeting of Hodgkin and Reed‐Sternberg Cells of Hodgkin's Disease With Monoclonal Antibody Ber‐H2 (CD30): Immunohistological Evidence,” British Journal of Haematology 82, no. 1 (1992): 38–45. 1329918 10.1111/j.1365-2141.1992.tb04591.x

[36] K. Kucka and H. Wajant , “Receptor Oligomerization and Its Relevance for Signaling by Receptors of the Tumor Necrosis Factor Receptor Superfamily,” Frontiers in Cell and Developmental Biology 8 (2020): 615141. 33644033 10.3389/fcell.2020.615141 PMC7905041

[37] L. M. Boucher , L. E. Marengere , Y. Lu , S. Thukral , and T. W. Mak , “Binding Sites of Cytoplasmic Effectors TRAF1, 2, and 3 on CD30 and Other Members of the TNF Receptor Superfamily,” Biochemical and Biophysical Research Communications 233, no. 3 (1997): 592–600. 9168896 10.1006/bbrc.1997.6509

[38] C. S. Duckett , R. W. Gedrich , M. C. Gilfillan , and C. B. Thompson , “Induction of Nuclear Factor kappaB by the CD30 Receptor Is Mediated by TRAF1 and TRAF2,” Molecular and Cellular Biology 17, no. 3 (1997): 1535–1542. 9032281 10.1128/mcb.17.3.1535 PMC231879

[39] N. Y. Thakar , D. A. Ovchinnikov , M. L. Hastie , B. Kobe , J. J. Gorman , and E. J. Wolvetang , “TRAF2 Recruitment via T61 in CD30 Drives NFkappaB Activation and Enhances hESC Survival and Proliferation,” Molecular Biology of the Cell 26, no. 5 (2015): 993–1006. 25568342 10.1091/mbc.E14-08-1290 PMC4342033

[40] B. Zheng , P. Fiumara , Y. V. Li , et al., “MEK/ERK Pathway Is Aberrantly Active in Hodgkin Disease: A Signaling Pathway Shared by CD30, CD40, and RANK That Regulates Cell Proliferation and Survival,” Blood 102, no. 3 (2003): 1019–1027. 12689928 10.1182/blood-2002-11-3507

[41] S. V. Krysov , T. F. Rowley , and A. Al‐Shamkhani , “Inhibition of p38 Mitogen‐Activated Protein Kinase Unmasks a CD30‐Triggered Apoptotic Pathway in Anaplastic Large Cell Lymphoma Cells,” Molecular Cancer Therapeutics 6, no. 2 (2007): 703–711. 17308066 10.1158/1535-7163.MCT-06-0544

[42] M. Watanabe , K. Nakano , T. Togano , et al., “Targeted Repression of Overexpressed CD30 Downregulates NF‐kappaB and ERK1/2 Pathway in Hodgkin Lymphoma Cell Lines,” Oncology Research 19, no. 10–11 (2011): 463–469. 22715589 10.3727/096504012x13285365944292

[43] R. Horie , T. Watanabe , Y. Morishita , et al., “Ligand‐Independent Signaling by Overexpressed CD30 Drives NF‐kappaB Activation in Hodgkin‐Reed‐Sternberg Cells,” Oncogene 21, no. 16 (2002): 2493–2503. 11971184 10.1038/sj.onc.1205337

[44] R. Horie , S. Aizawa , M. Nagai , et al., “A Novel Domain in the CD30 Cytoplasmic Tail Mediates NFkappaB Activation,” International Immunology 10, no. 2 (1998): 203–210. 9533448 10.1093/intimm/10.2.203

[45] B. Hirsch , M. Hummel , S. Bentink , et al., “CD30‐Induced Signaling Is Absent in Hodgkin's Cells but Present in Anaplastic Large Cell Lymphoma Cells,” American Journal of Pathology 172, no. 2 (2008): 510–520. 18187570 10.2353/ajpath.2008.070858 PMC2312360

[46] M. Nishikori , H. Ohno , H. Haga , and T. Uchiyama , “Stimulation of CD30 in Anaplastic Large Cell Lymphoma Leads to Production of Nuclear Factor‐kappaB p52, Which Is Associated With Hyperphosphorylated Bcl‐3,” Cancer Science 96, no. 8 (2005): 487–497. 16108830 10.1111/j.1349-7006.2005.00078.x PMC11159099

[47] M. Watanabe , M. Sasaki , K. Itoh , et al., “JunB Induced by Constitutive CD30‐Extracellular Signal‐Regulated Kinase 1/2 Mitogen‐Activated Protein Kinase Signaling Activates the CD30 Promoter in Anaplastic Large Cell Lymphoma and Reed‐Sternberg Cells of Hodgkin Lymphoma,” Cancer Research 65, no. 17 (2005): 7628–7634. 16140928 10.1158/0008-5472.CAN-05-0925

[48] R. L. Boddicker , N. S. Kip , X. Xing , et al., “The Oncogenic Transcription Factor IRF4 Is Regulated by a Novel CD30/NF‐kappaB Positive Feedback Loop in Peripheral T‐Cell Lymphoma,” Blood 125, no. 20 (2015): 3118–3127. 25833963 10.1182/blood-2014-05-578575 PMC4432006

[49] A. Pham‐Ledard , M. Prochazkova‐Carlotti , E. Laharanne , et al., “IRF4 Gene Rearrangements Define a Subgroup of CD30‐Positive Cutaneous T‐Cell Lymphoma: A Study of 54 Cases,” Journal of Investigative Dermatology 130, no. 3 (2010): 816–825. 19812605 10.1038/jid.2009.314

[50] M. Watanabe , K. Itoh , T. Togano , et al., “Ets‐1 Activates Overexpression of JunB and CD30 in Hodgkin's Lymphoma and Anaplastic Large‐Cell Lymphoma,” American Journal of Pathology 180, no. 2 (2012): 831–838. 22107829 10.1016/j.ajpath.2011.10.007 PMC4429177

[51] M. Watanabe , Y. Ogawa , K. Ito , et al., “AP‐1 Mediated Relief of Repressive Activity of the CD30 Promoter Microsatellite in Hodgkin and Reed‐Sternberg Cells,” American Journal of Pathology 163, no. 2 (2003): 633–641. 12875982 10.1016/S0002-9440(10)63690-5 PMC1868231

[52] H. Kanzler , R. Kuppers , M. L. Hansmann , and K. Rajewsky , “Hodgkin and Reed‐Sternberg Cells in Hodgkin's Disease Represent the Outgrowth of a Dominant Tumor Clone Derived From (Crippled) Germinal Center B Cells,” Journal of Experimental Medicine 184, no. 4 (1996): 1495–1505. 8879220 10.1084/jem.184.4.1495 PMC2192840

[53] R. Kuppers , “Advances in Hodgkin Lymphoma Research,” Trends in Molecular Medicine 31, no. 4 (2025): 326–343. 39443214 10.1016/j.molmed.2024.10.004

[54] R. Kuppers , H. Kanzler , M. L. Hansmann , and K. Rajewsky , “Immunoglobulin V Genes in Reed‐Sternberg Cells,” New England Journal of Medicine 334, no. 6 (1996): 404–406. 10.1056/NEJM199602083340615 8538724

[55] R. Kuppers , K. Rajewsky , M. Zhao , et al., “Hodgkin Disease: Hodgkin and Reed‐Sternberg Cells Picked From Histological Sections Show Clonal Immunoglobulin Gene Rearrangements and Appear to Be Derived From B Cells at Various Stages of Development,” Proceedings of the National Academy of Sciences of the United States of America 91, no. 23 (1994): 10962–10966. 7971992 10.1073/pnas.91.23.10962 PMC45146

[56] T. Marafioti , M. Hummel , H. D. Foss , et al., “Hodgkin and Reed‐Sternberg Cells Represent an Expansion of a Single Clone Originating From a Germinal Center B‐Cell With Functional Immunoglobulin Gene Rearrangements but Defective Immunoglobulin Transcription,” Blood 95, no. 4 (2000): 1443–1450. 10666223

[57] H. D. Foss , R. Reusch , G. Demel , et al., “Frequent Expression of the B‐Cell‐Specific Activator Protein in Reed‐Sternberg Cells of Classical Hodgkin's Disease Provides Further Evidence for Its B‐Cell Origin,” Blood 94, no. 9 (1999): 3108–3113. 10556196

[58] J. Gerdes , R. Schwarting , and H. Stein , “High Proliferative Activity of Reed Sternberg Associated Antigen Ki‐1 Positive Cells in Normal Lymphoid Tissue,” Journal of Clinical Pathology 39, no. 9 (1986): 993–997. 3020097 10.1136/jcp.39.9.993 PMC500199

[59] M. A. Weniger , E. Tiacci , S. Schneider , et al., “Human CD30+ B Cells Represent a Unique Subset Related to Hodgkin Lymphoma Cells,” Journal of Clinical Investigation 128, no. 7 (2018): 2996–3007. 29889102 10.1172/JCI95993 PMC6025985

[60] R. Kuppers , B. Budeus , S. Hartmann , and M. L. Hansmann , “Clonal Composition and Differentiation Stage of Human CD30(+) B Cells in Reactive Lymph Nodes,” Frontiers in Immunology 14 (2023): 1208610. 37559724 10.3389/fimmu.2023.1208610 PMC10407394

[61] L. A. Julien , R. P. Michel , and M. Auger , “Breast Implant‐Associated Anaplastic Large Cell Lymphoma and Effusions: A Review With Emphasis on the Role of Cytopathology,” Cancer Cytopathology 128, no. 7 (2020): 440–451. 31899606 10.1002/cncy.22233

[62] H. Stein , H. D. Foss , H. Durkop , et al., “CD30(+) Anaplastic Large Cell Lymphoma: A Review of Its Histopathologic, Genetic, and Clinical Features,” Blood 96, no. 12 (2000): 3681–3695. 11090048

[63] S. Pileri , B. Falini , G. Delsol , et al., “Lymphohistiocytic T‐Cell Lymphoma (Anaplastic Large Cell Lymphoma CD30+/Ki‐1 + With a High Content of Reactive Histiocytes),” Histopathology 16, no. 4 (1990): 383–391. 2163351 10.1111/j.1365-2559.1990.tb01143.x

[64] M. Piris , D. C. Brown , K. C. Gatter , and D. Y. Mason , “CD30 Expression in Non‐Hodgkin's Lymphoma,” Histopathology 17, no. 3 (1990): 211–218. 2173674 10.1111/j.1365-2559.1990.tb00709.x

[65] B. Falini , S. Pileri , P. L. Zinzani , et al., “ALK+ Lymphoma: Clinico‐Pathological Findings and Outcome,” Blood 93, no. 8 (1999): 2697–2706. 10194450

[66] G. Pallesen , “The Diagnostic Significance of the CD30 (Ki‐1) Antigen,” Histopathology 16, no. 4 (1990): 409–413. 2163354 10.1111/j.1365-2559.1990.tb01151.x

[67] A. Traverse‐Glehen , S. Pittaluga , P. Gaulard , et al., “Mediastinal Gray Zone Lymphoma: The Missing Link Between Classic Hodgkin's Lymphoma and Mediastinal Large B‐Cell Lymphoma,” American Journal of Surgical Pathology 29, no. 11 (2005): 1411–1421. 16224207 10.1097/01.pas.0000180856.74572.73

[68] J. P. Higgins and R. A. Warnke , “CD30 Expression Is Common in Mediastinal Large B‐Cell Lymphoma,” American Journal of Clinical Pathology 112, no. 2 (1999): 241–247. 10439805 10.1093/ajcp/112.2.241

[69] W. H. Wilson , S. Pittaluga , A. Nicolae , et al., “A Prospective Study of Mediastinal Gray‐Zone Lymphoma,” Blood 124, no. 10 (2014): 1563–1569. 25024303 10.1182/blood-2014-03-564906 PMC4155269

[70] S. Hartmann , O. Goncharova , A. Portyanko , et al., “CD30 Expression in Neoplastic T Cells of Follicular T Cell Lymphoma Is a Helpful Diagnostic Tool in the Differential Diagnosis of Hodgkin Lymphoma,” Modern Pathology 32, no. 1 (2019): 37–47. 30140037 10.1038/s41379-018-0108-5

[71] S. L. Abbondanzo , N. Sato , S. E. Straus , and E. S. Jaffe , “Acute Infectious Mononucleosis. CD30 (Ki‐1) Antigen Expression and Histologic Correlations,” American Journal of Clinical Pathology 93, no. 5 (1990): 698–702. 2158227 10.1093/ajcp/93.5.698

[72] E. Campo , E. S. Jaffe , J. R. Cook , et al., “The International Consensus Classification of Mature Lymphoid Neoplasms: A Report From the Clinical Advisory Committee,” Blood 140, no. 11 (2022): 1229–1253. 35653592 10.1182/blood.2022015851 PMC9479027

[73] G. Pallesen and S. J. Hamilton‐Dutoit , “Ki‐1 (CD30) Antigen Is Regularly Expressed by Tumor Cells of Embryonal Carcinoma,” American Journal of Pathology 133, no. 3 (1988): 446–450. 2849300 PMC1880812

[74] U. Latza , H. D. Foss , H. Durkop , et al., “CD30 Antigen in Embryonal Carcinoma and Embryogenesis and Release of the Soluble Molecule,” American Journal of Pathology 146, no. 2 (1995): 463–471. 7856755 PMC1869849

[75] K. Blatt , S. Cerny‐Reiterer , J. Schwaab , et al., “Identification of the Ki‐1 Antigen (CD30) as a Novel Therapeutic Target in Systemic Mastocytosis,” Blood 126, no. 26 (2015): 2832–2841. 26486787 10.1182/blood-2015-03-637728 PMC4692143

[76] A. Iwakoshi , H. Kikui , R. Nakashima , et al., “CD30 Expression in an Emerging Group of Mesenchymal Spindle Cell Neoplasms With ALK Fusion Detected by Flow Cytometry and Immunohistochemistry,” Genes, Chromosomes & Cancer 63, no. 2 (2024): e23228. 38380728 10.1002/gcc.23228

[77] H. Durkop , H. D. Foss , F. Eitelbach , et al., “Expression of the CD30 Antigen in Non‐Lymphoid Tissues and Cells,” Journal of Pathology 190, no. 5 (2000): 613–618. 10727988 10.1002/(SICI)1096-9896(200004)190:5<613::AID-PATH559>3.0.CO;2-0

[78] P. L. Tazzari , A. Bolognesi , D. de Totero , et al., “Ber‐H2 (Anti‐CD30)‐Saporin Immunotoxin: A New Tool for the Treatment of Hodgkin's Disease and CD30+ Lymphoma: In Vitro Evaluation,” British Journal of Haematology 81, no. 2 (1992): 203–211. 1322690 10.1111/j.1365-2141.1992.tb08208.x

[79] A. F. Wahl , K. Klussman , J. D. Thompson , et al., “The Anti‐CD30 Monoclonal Antibody SGN‐30 Promotes Growth Arrest and DNA Fragmentation In Vitro and Affects Antitumor Activity in Models of Hodgkin's Disease,” Cancer Research 62, no. 13 (2002): 3736–3742. 12097283

[80] C. G. Cerveny , C. L. Law , R. S. McCormick , et al., “Signaling via the Anti‐CD30 mAb SGN‐30 Sensitizes Hodgkin's Disease Cells to Conventional Chemotherapeutics,” Leukemia 19, no. 9 (2005): 1648–1655. 16049514 10.1038/sj.leu.2403884

[81] P. Borchmann , J. F. Treml , H. Hansen , et al., “The Human Anti‐CD30 Antibody 5F11 Shows In Vitro and In Vivo Activity Against Malignant Lymphoma,” Blood 102, no. 10 (2003): 3737–3742. 12881320 10.1182/blood-2003-02-0515

[82] N. L. Bartlett , A. Younes , M. H. Carabasi , et al., “A Phase 1 Multidose Study of SGN‐30 Immunotherapy in Patients With Refractory or Recurrent CD30+ Hematologic Malignancies,” Blood 111, no. 4 (2008): 1848–1854. 18079362 10.1182/blood-2008-01-127118 PMC2275000

[83] A. Forero‐Torres , J. P. Leonard , A. Younes , et al., “A Phase II Study of SGN‐30 (Anti‐CD30 mAb) in Hodgkin Lymphoma or Systemic Anaplastic Large Cell Lymphoma,” British Journal of Haematology 146, no. 2 (2009): 171–179. 19466965 10.1111/j.1365-2141.2009.07740.x

[84] S. M. Ansell , S. M. Horwitz , A. Engert , et al., “Phase I/II Study of an Anti‐CD30 Monoclonal Antibody (MDX‐060) in Hodgkin's Lymphoma and Anaplastic Large‐Cell Lymphoma,” Journal of Clinical Oncology 25, no. 19 (2007): 2764–2769. 17515574 10.1200/JCO.2006.07.8972

[85] L. Pasqualucci , M. Wasik , B. A. Teicher , et al., “Antitumor Activity of Anti‐CD30 Immunotoxin (Ber‐H2/Saporin) In Vitro and in Severe Combined Immunodeficiency Disease Mice Xenografted With Human CD30+ Anaplastic Large‐Cell Lymphoma,” Blood 85, no. 8 (1995): 2139–2146. 7718885

[86] A. Terenzi , A. Bolognesi , L. Pasqualucci , et al., “Anti‐CD30 (BER=H2) Immunotoxins Containing the Type‐1 Ribosome‐Inactivating Proteins Momordin and PAP‐S (Pokeweed Antiviral Protein From Seeds) Display Powerful Antitumour Activity Against CD30+ Tumour Cells In Vitro and in SCID Mice,” British Journal of Haematology 92, no. 4 (1996): 872–879. 8616080 10.1046/j.1365-2141.1995.404942.x

[87] B. Falini , A. Bolognesi , L. Flenghi , et al., “Response of Refractory Hodgkin's Disease to Monoclonal Anti‐CD30 Immunotoxin,” Lancet 339, no. 8803 (1992): 1195–1196. 1349939 10.1016/0140-6736(92)91135-u

[88] R. Schnell , O. Staak , P. Borchmann , et al., “A Phase I Study With an Anti‐CD30 Ricin A‐Chain Immunotoxin (Ki‐4.dgA) in Patients With Refractory CD30+ Hodgkin's and Non‐Hodgkin's Lymphoma,” Clinical Cancer Research 8, no. 6 (2002): 1779–1786. 12060617

[89] J. A. Francisco , C. G. Cerveny , D. L. Meyer , et al., “cAC10‐vcMMAE, an Anti‐CD30‐Monomethyl Auristatin E Conjugate With Potent and Selective Antitumor Activity,” Blood 102, no. 4 (2003): 1458–1465. 12714494 10.1182/blood-2003-01-0039

[90] S. O. Doronina , B. E. Toki , M. Y. Torgov , et al., “Development of Potent Monoclonal Antibody Auristatin Conjugates for Cancer Therapy,” Nature Biotechnology 21, no. 7 (2003): 778–784. 10.1038/nbt832 12778055

[91] N. M. Okeley , J. B. Miyamoto , X. Zhang , et al., “Intracellular Activation of SGN‐35, a Potent Anti‐CD30 Antibody‐Drug Conjugate,” Clinical Cancer Research 16, no. 3 (2010): 888–897. 20086002 10.1158/1078-0432.CCR-09-2069

[92] M. S. Sutherland , R. J. Sanderson , K. A. Gordon , et al., “Lysosomal Trafficking and Cysteine Protease Metabolism Confer Target‐Specific Cytotoxicity by Peptide‐Linked Anti‐CD30‐Auristatin Conjugates,” Journal of Biological Chemistry 281, no. 15 (2006): 10540–10547. 16484228 10.1074/jbc.M510026200

[93] P. Muller , K. Martin , S. Theurich , et al., “Microtubule‐Depolymerizing Agents Used in Antibody‐Drug Conjugates Induce Antitumor Immunity by Stimulation of Dendritic Cells,” Cancer Immunology Research 2, no. 8 (2014): 741–755. 24916470 10.1158/2326-6066.CIR-13-0198

[94] C. Burton , P. Allen , and A. F. Herrera , “Paradigm Shifts in Hodgkin Lymphoma Treatment: From Frontline Therapies to Relapsed Disease,” American Society of Clinical Oncology Educational Book 44, no. 3 (2024): e433502. 38728605 10.1200/EDBK_433502

[95] C. Benevolo Savelli , M. Bisio , L. Legato , et al., “Advances in Hodgkin Lymphoma Treatment: From Molecular Biology to Clinical Practice,” Cancers 16, no. 10 (2024): 1830. 38791909 10.3390/cancers16101830 PMC11120540

[96] R. Chen , A. F. Herrera , J. Hou , et al., “Inhibition of MDR1 Overcomes Resistance to Brentuximab Vedotin in Hodgkin Lymphoma,” Clinical Cancer Research 26, no. 5 (2020): 1034–1044. 31811017 10.1158/1078-0432.CCR-19-1768 PMC7056527

[97] R. Chen , J. Hou , E. Newman , et al., “CD30 Downregulation, MMAE Resistance, and MDR1 Upregulation Are All Associated With Resistance to Brentuximab Vedotin,” Molecular Cancer Therapeutics 14, no. 6 (2015): 1376–1384. 25840583 10.1158/1535-7163.MCT-15-0036 PMC4458438

[98] H. P. Hansen , A. Trad , M. Dams , et al., “CD30 on Extracellular Vesicles From Malignant Hodgkin Cells Supports Damaging of CD30 Ligand‐Expressing Bystander Cells With Brentuximab‐Vedotin, In Vitro,” Oncotarget 7, no. 21 (2016): 30523–30535. 27105521 10.18632/oncotarget.8864 PMC5058698

[99] N. Nathwani , A. Y. Krishnan , Q. Huang , et al., “Persistence of CD30 Expression in Hodgkin Lymphoma Following Brentuximab Vedotin (SGN‐35) Treatment Failure,” Leukemia & Lymphoma 53, no. 10 (2012): 2051–2053. 22369501 10.3109/10428194.2012.666543 PMC3808117

[100] C. Nielson , R. Fischer , G. Fraga , and D. Aires , “Loss of CD30 Expression in Anaplastic Large Cell Lymphoma Following Brentuximab Therapy,” Journal of Drugs in Dermatology 15, no. 7 (2016): 894–895. 27391642

[101] R. N. Al‐Rohil , C. A. Torres‐Cabala , A. Patel , et al., “Loss of CD30 Expression After Treatment With Brentuximab Vedotin in a Patient With Anaplastic Large Cell Lymphoma: A Novel Finding,” Journal of Cutaneous Pathology 43, no. 12 (2016): 1161–1166. 27531242 10.1111/cup.12797

[102] Y. Kaimi , Y. Takahashi , H. Taniguchi , et al., “Loss of or Decrease in CD30 Expression in Four Patients With Anaplastic Large Cell Lymphoma After Brentuximab Vedotin‐Containing Therapy,” Virchows Archiv 484, no. 3 (2024): 465–473. 38349387 10.1007/s00428-024-03764-1

[103] E. J. Dann and R. O. Casasnovas , “Treatment Strategies in Advanced‐Stage Hodgkin Lymphoma,” Cancers 16, no. 11 (2024): 2059. 38893177 10.3390/cancers16112059 PMC11171059

[104] J. M. Connors , W. Jurczak , D. J. Straus , et al., “Brentuximab Vedotin With Chemotherapy for Stage III or IV Hodgkin's Lymphoma,” New England Journal of Medicine 378, no. 4 (2018): 331–344. 29224502 10.1056/NEJMoa1708984 PMC5819601

[105] J. G. Schroers‐Martin and R. Advani , “When Should We Use It? The Role of Brentuximab Vedotin in 2024,” Hematology. American Society of Hematology. Education Program 2024, no. 1 (2024): 511–516. 39644065 10.1182/hematology.2024000668 PMC11665589

[106] S. M. Ansell , J. Radford , J. M. Connors , et al., “Overall Survival With Brentuximab Vedotin in Stage III or IV Hodgkin's Lymphoma,” New England Journal of Medicine 387, no. 4 (2022): 310–320. 35830649 10.1056/NEJMoa2206125

[107] D. A. Eichenauer , A. Plutschow , S. Kreissl , et al., “Incorporation of Brentuximab Vedotin Into First‐Line Treatment of Advanced Classical Hodgkin's Lymphoma: Final Analysis of a Phase 2 Randomised Trial by the German Hodgkin Study Group,” Lancet Oncology 18, no. 12 (2017): 1680–1687. 29133014 10.1016/S1470-2045(17)30696-4

[108] C. Damaschin , H. Goergen , S. Kreissl , et al., “Brentuximab Vedotin‐Containing Escalated BEACOPP Variants for Newly Diagnosed Advanced‐Stage Classical Hodgkin Lymphoma: Follow‐Up Analysis of a Randomized Phase II Study From the German Hodgkin Study Group,” Leukemia 36, no. 2 (2022): 580–582. 34408266 10.1038/s41375-021-01386-z PMC8807388

[109] P. Borchmann , J. Ferdinandus , G. Schneider , et al., “Assessing the Efficacy and Tolerability of PET‐Guided BrECADD Versus eBEACOPP in Advanced‐Stage, Classical Hodgkin Lymphoma (HD21): A Randomised, Multicentre, Parallel, Open‐Label, Phase 3 Trial,” Lancet 404, no. 10450 (2024): 341–352. 38971175 10.1016/S0140-6736(24)01315-1

[110] A. M. Evens , F. Hong , L. I. Gordon , et al., “The Efficacy and Tolerability of Adriamycin, Bleomycin, Vinblastine, Dacarbazine and Stanford V in Older Hodgkin Lymphoma Patients: A Comprehensive Analysis From the North American Intergroup Trial E2496,” British Journal of Haematology 161, no. 1 (2013): 76–86. 23356491 10.1111/bjh.12222 PMC3906856

[111] A. Fossa , D. Molin , P. J. Brockelmann , et al., “Brentuximab Vedotin Monotherapy Is a Feasible and Effective Treatment for Older Patients With Classical Hodgkin Lymphoma Unsuitable for Curative Chemotherapy: Results From the Prospective GHSG‐NLG Phase II BVB Trial,” Hema 9, no. 3 (2025): e70099. 10.1002/hem3.70099 PMC11926767 40124718

[112] A. Forero‐Torres , B. Holkova , J. Goldschmidt , et al., “Phase 2 Study of Frontline Brentuximab Vedotin Monotherapy in Hodgkin Lymphoma Patients Aged 60 Years and Older,” Blood 126, no. 26 (2015): 2798–2804. 26377597 10.1182/blood-2015-06-644336 PMC4692140

[113] A. M. Evens , R. H. Advani , I. B. Helenowski , et al., “Multicenter Phase II Study of Sequential Brentuximab Vedotin and Doxorubicin, Vinblastine, and Dacarbazine Chemotherapy for Older Patients With Untreated Classical Hodgkin Lymphoma,” Journal of Clinical Oncology 36, no. 30 (2018): 3015–3022. 30179569 10.1200/JCO.2018.79.0139

[114] J. W. Friedberg , R. Bordoni , D. Patel‐Donnelly , et al., “Brentuximab Vedotin With Dacarbazine or Nivolumab as Frontline cHL Therapy for Older Patients Ineligible for Chemotherapy,” Blood 143, no. 9 (2024): 786–795. 37946283 10.1182/blood.2022019536

[115] B. D. Cheson , N. L. Bartlett , B. Knopf , et al., “Brentuximab Vedotin and Nivolumab for Untreated Patients With Hodgkin Lymphoma: Long‐Term Results,” Blood Advances 9, no. 15 (2025): 3750–3753. 40311072 10.1182/bloodadvances.2025016470 PMC12305559

[116] P. G. Rubinstein , P. C. Moore , M. A. Rudek , et al., “Brentuximab Vedotin With AVD Shows Safety, in the Absence of Strong CYP3A4 Inhibitors, in Newly Diagnosed HIV‐Associated Hodgkin Lymphoma,” Aids 32, no. 5 (2018): 605–611. 29280762 10.1097/QAD.0000000000001729 PMC5832596

[117] P. G. Rubinstein , P. C. Moore , M. Bimali , et al., “Brentuximab Vedotin With AVD for Stage II‐IV HIV‐Related Hodgkin Lymphoma (AMC 085): Phase 2 Results From an Open‐Label, Single Arm, Multicentre Phase 1/2 Trial,” Lancet Haematology 10, no. 8 (2023): e624–e632. 37532416 10.1016/S2352-3026(23)00157-6 PMC10859222

[118] L. M. Fornecker , J. Lazarovici , I. Aurer , et al., “Brentuximab Vedotin Plus AVD for First‐Line Treatment of Early‐Stage Unfavorable Hodgkin Lymphoma (BREACH): A Multicenter, Open‐Label, Randomized, Phase II Trial,” Journal of Clinical Oncology 41, no. 2 (2023): 327–335. 35867960 10.1200/JCO.21.01281

[119] A. Kumar , C. Casulo , J. Yahalom , et al., “Brentuximab Vedotin and AVD Followed by Involved‐Site Radiotherapy in Early Stage, Unfavorable Risk Hodgkin Lymphoma,” Blood 128, no. 11 (2016): 1458–1464. 27458003 10.1182/blood-2016-03-703470 PMC5025897

[120] S. I. Park , T. C. Shea , O. Olajide , et al., “ABVD Followed by BV Consolidation in Risk‐Stratified Patients With Limited‐Stage Hodgkin Lymphoma,” Blood Advances 4, no. 11 (2020): 2548–2555. 32516414 10.1182/bloodadvances.2020001871 PMC7284091

[121] J. S. Abramson , J. E. Arnason , A. S. LaCasce , et al., “Brentuximab Vedotin, Doxorubicin, Vinblastine, and Dacarbazine for Nonbulky Limited‐Stage Classical Hodgkin Lymphoma,” Blood 134, no. 7 (2019): 606–613. 31186274 10.1182/blood.2019001272

[122] J. S. Abramson , E. Bengston , R. Redd , et al., “Brentuximab Vedotin Plus Doxorubicin and Dacarbazine in Nonbulky Limited‐Stage Classical Hodgkin Lymphoma,” Blood Advances 7, no. 7 (2023): 1130–1136. 36053786 10.1182/bloodadvances.2022008420 PMC10111342

[123] A. Younes , N. L. Bartlett , J. P. Leonard , et al., “Brentuximab Vedotin (SGN‐35) for Relapsed CD30‐Positive Lymphomas,” New England Journal of Medicine 363, no. 19 (2010): 1812–1821. 21047225 10.1056/NEJMoa1002965

[124] R. Chen , A. K. Gopal , S. E. Smith , et al., “Five‐Year Survival and Durability Results of Brentuximab Vedotin in Patients With Relapsed or Refractory Hodgkin Lymphoma,” Blood 128, no. 12 (2016): 1562–1566. 27432875 10.1182/blood-2016-02-699850 PMC5034737

[125] A. Younes , A. K. Gopal , S. E. Smith , et al., “Results of a Pivotal Phase II Study of Brentuximab Vedotin for Patients With Relapsed or Refractory Hodgkin's Lymphoma,” Journal of Clinical Oncology 30, no. 18 (2012): 2183–2189. 22454421 10.1200/JCO.2011.38.0410 PMC3646316

[126] A. F. Herrera , J. Palmer , P. Martin , et al., “Autologous Stem‐Cell Transplantation After Second‐Line Brentuximab Vedotin in Relapsed or Refractory Hodgkin Lymphoma,” Annals of Oncology 29, no. 3 (2018): 724–730. 29272364 10.1093/annonc/mdx791 PMC5889038

[127] R. Chen , J. M. Palmer , P. Martin , et al., “Results of a Multicenter Phase II Trial of Brentuximab Vedotin as Second‐Line Therapy Before Autologous Transplantation in Relapsed/Refractory Hodgkin Lymphoma,” Biology of Blood and Marrow Transplantation 21, no. 12 (2015): 2136–2140. 26211987 10.1016/j.bbmt.2015.07.018 PMC4639410

[128] R. H. Advani , A. J. Moskowitz , N. L. Bartlett , et al., “Brentuximab Vedotin in Combination With Nivolumab in Relapsed or Refractory Hodgkin Lymphoma: 3‐Year Study Results,” Blood 138, no. 6 (2021): 427–438. 33827139 10.1182/blood.2020009178 PMC12684812

[129] A. J. Moskowitz , H. Schoder , J. Yahalom , et al., “PET‐Adapted Sequential Salvage Therapy With Brentuximab Vedotin Followed by Augmented Ifosamide, Carboplatin, and Etoposide for Patients With Relapsed and Refractory Hodgkin's Lymphoma: A Non‐Randomised, Open‐Label, Single‐Centre, Phase 2 Study,” Lancet Oncology 16, no. 3 (2015): 284–292. 25683846 10.1016/S1470-2045(15)70013-6

[130] R. C. Lynch , R. D. Cassaday , S. D. Smith , et al., “Dose‐Dense Brentuximab Vedotin Plus Ifosfamide, Carboplatin, and Etoposide for Second‐Line Treatment of Relapsed or Refractory Classical Hodgkin Lymphoma: A Single Centre, Phase 1/2 Study,” Lancet Haematology 8, no. 8 (2021): e562–e571. 34329577 10.1016/S2352-3026(21)00170-8 PMC8457616

[131] R. Garcia‐Sanz , A. Sureda , F. de la Cruz , et al., “Brentuximab Vedotin and ESHAP Is Highly Effective as Second‐Line Therapy for Hodgkin Lymphoma Patients (Long‐Term Results of a Trial by the Spanish GELTAMO Group),” Annals of Oncology 30, no. 4 (2019): 612–620. 30657848 10.1093/annonc/mdz009

[132] M. J. Kersten , J. Driessen , J. M. Zijlstra , et al., “Combining Brentuximab Vedotin With Dexamethasone, High‐Dose Cytarabine and Cisplatin as Salvage Treatment in Relapsed or Refractory Hodgkin Lymphoma: The Phase II HOVON/LLPC Transplant BRaVE Study,” Haematologica 106, no. 4 (2021): 1129–1137. 32273476 10.3324/haematol.2019.243238 PMC8018114

[133] A. S. LaCasce , R. G. Bociek , A. Sawas , et al., “Three‐Year Outcomes With Brentuximab Vedotin Plus Bendamustine as First Salvage Therapy in Relapsed or Refractory Hodgkin Lymphoma,” British Journal of Haematology 189, no. 3 (2020): e86–e90. 32048731 10.1111/bjh.16499

[134] J. Driessen , F. de Wit , A. F. Herrera , et al., “Brentuximab Vedotin and Chemotherapy in Relapsed/Refractory Hodgkin Lymphoma: A Propensity Score‐Matched Analysis,” Blood Advances 8, no. 11 (2024): 2740–2752. 38502227 10.1182/bloodadvances.2023012145 PMC11170165

[135] A. Sureda , A. Pavlovsky , D. Haidar , F. Kristo , V. Stache , and A. Zomas , “Real‐World Outcomes of Brentuximab Vedotin as Consolidation Therapy After Autologous Stem Cell Transplantation in Relapsed/Refractory Hodgkin Lymphoma: A Systematic Review and Meta‐Analysis,” Bone Marrow Transplantation 60, no. 6 (2025): 820–831. 40200006 10.1038/s41409-025-02557-7 PMC12151861

[136] C. H. Moskowitz , A. Nademanee , T. Masszi , et al., “Brentuximab Vedotin as Consolidation Therapy After Autologous Stem‐Cell Transplantation in Patients With Hodgkin's Lymphoma at Risk of Relapse or Progression (AETHERA): A Randomised, Double‐Blind, Placebo‐Controlled, Phase 3 Trial,” Lancet 385, no. 9980 (2015): 1853–1862. 25796459 10.1016/S0140-6736(15)60165-9

[137] C. H. Moskowitz , J. Walewski , A. Nademanee , et al., “Five‐Year PFS From the AETHERA Trial of Brentuximab Vedotin for Hodgkin Lymphoma at High Risk of Progression or Relapse,” Blood 132, no. 25 (2018): 2639–2642. 30266774 10.1182/blood-2018-07-861641

[138] H. S. Ngu and K. J. Savage , “Frontline Management of Nodal Peripheral T‐Cell Lymphomas,” American Society of Clinical Oncology Educational Book 43 (2023): e390334. 37262395 10.1200/EDBK_390334

[139] F. Ellin , J. Landstrom , M. Jerkeman , and T. Relander , “Real‐World Data on Prognostic Factors and Treatment in Peripheral T‐Cell Lymphomas: A Study From the Swedish Lymphoma Registry,” Blood 124, no. 10 (2014): 1570–1577. 25006130 10.1182/blood-2014-04-573089

[140] S. K. Barta , J. Z. Gong , and P. Porcu , “Brentuximab Vedotin in the Treatment of CD30+ PTCL,” Blood 134, no. 26 (2019): 2339–2345. 31697814 10.1182/blood.2019001821

[141] M. A. Fanale , S. M. Horwitz , A. Forero‐Torres , et al., “Brentuximab Vedotin in the Front‐Line Treatment of Patients With CD30+ Peripheral T‐Cell Lymphomas: Results of a Phase I Study,” Journal of Clinical Oncology 32, no. 28 (2014): 3137–3143. 25135998 10.1200/JCO.2013.54.2456 PMC4171358

[142] M. A. Fanale , S. M. Horwitz , A. Forero‐Torres , et al., “Five‐Year Outcomes for Frontline Brentuximab Vedotin With CHP for CD30‐Expressing Peripheral T‐Cell Lymphomas,” Blood 131, no. 19 (2018): 2120–2124. 29507077 10.1182/blood-2017-12-821009 PMC5946765

[143] S. Horwitz , O. A. O'Connor , B. Pro , et al., “Brentuximab Vedotin With Chemotherapy for CD30‐Positive Peripheral T‐Cell Lymphoma (ECHELON‐2): A Global, Double‐Blind, Randomised, Phase 3 Trial,” Lancet 393, no. 10168 (2019): 229–240. 30522922 10.1016/S0140-6736(18)32984-2 PMC6436818

[144] S. Horwitz , O. A. O'Connor , B. Pro , et al., “The ECHELON‐2 Trial: 5‐Year Results of a Randomized, Phase III Study of Brentuximab Vedotin With Chemotherapy for CD30‐Positive Peripheral T‐Cell Lymphoma,” Annals of Oncology 33, no. 3 (2022): 288–298. 34921960 10.1016/j.annonc.2021.12.002 PMC9447792

[145] E. Domingo‐Domenech , B. Pro , T. Illidge , et al., “Brentuximab Vedotin Plus Chemotherapy for the Treatment of Front‐Line Systemic Anaplastic Large Cell Lymphoma: Subgroup Analysis of the ECHELON‐2 Study at 5 Years' Follow‐Up,” Blood Cancer Journal 15, no. 1 (2025): 129. 40750774 10.1038/s41408-025-01329-2 PMC12317119

[146] K. J. Savage , S. M. Horwitz , R. Advani , et al., “Role of Stem Cell Transplant in CD30+ PTCL Following Frontline Brentuximab Vedotin Plus CHP or CHOP in ECHELON‐2,” Blood Advances 6, no. 19 (2022): 5550. 35470385 10.1182/bloodadvances.2020003971 PMC9647727

[147] A. F. Herrera , J. Zain , K. J. Savage , et al., “Brentuximab Vedotin Plus Cyclophosphamide, Doxorubicin, Etoposide, and Prednisone (CHEP‐BV) Followed by BV Consolidation in Patients With CD30‐Expressing Peripheral T‐Cell Lymphomas,” Blood 138 (2021): 133.

[148] A. F. Herrera , J. Zain , K. J. Savage , et al., “Brentuximab Vedotin Plus Cyclophosphamide, Doxorubicin, Etoposide, and Prednisone Followed by Brentuximab Vedotin Consolidation in CD30‐Positive Peripheral T‐Cell Lymphomas: A Multicentre, Single‐Arm, Phase 2 Study,” Lancet Haematology 11, no. 9 (2024): e671–e681. 39067464 10.1016/S2352-3026(24)00171-6

[149] C. Dittus , M. J. Weinstock , J. Barnes , et al., “Final Results of a Multicentre Pilot Study Evaluating Brentuximab Vedotin With Cyclophosphamide, Doxorubicin, Etoposide and Prednisone (BV‐CHEP) for the Treatment of Aggressive Adult T‐Cell Leukaemia/Lymphoma,” British Journal of Haematology 207 (2025): 299–303, 10.1111/bjh.20177. 40459047

[150] M. Duvic , M. T. Tetzlaff , P. Gangar , A. L. Clos , D. Sui , and R. Talpur , “Results of a Phase II Trial of Brentuximab Vedotin for CD30+ Cutaneous T‐Cell Lymphoma and Lymphomatoid Papulosis,” Journal of Clinical Oncology 33, no. 32 (2015): 3759–3765. 26261247 10.1200/JCO.2014.60.3787 PMC4737859

[151] H. M. Prince , Y. H. Kim , S. M. Horwitz , et al., “Brentuximab Vedotin or Physician's Choice in CD30‐Positive Cutaneous T‐Cell Lymphoma (ALCANZA): An International, Open‐Label, Randomised, Phase 3, Multicentre Trial,” Lancet 390, no. 10094 (2017): 555–566. 28600132 10.1016/S0140-6736(17)31266-7

[152] K. J. Savage , N. L. Harris , J. M. Vose , et al., “ALK‐ Anaplastic Large‐Cell Lymphoma Is Clinically and Immunophenotypically Different From Both ALK+ ALCL and Peripheral T‐Cell Lymphoma, Not Otherwise Specified: Report From the International Peripheral T‐Cell Lymphoma Project,” Blood 111, no. 12 (2008): 5496–5504. 18385450 10.1182/blood-2008-01-134270

[153] S. M. Smith , L. J. Burns , K. van Besien , et al., “Hematopoietic Cell Transplantation for Systemic Mature T‐Cell Non‐Hodgkin Lymphoma,” Journal of Clinical Oncology 31, no. 25 (2013): 3100–3109. 23897963 10.1200/JCO.2012.46.0188 PMC3753702

[154] A. Morel , J. Briere , L. Lamant , et al., “Long‐Term Outcomes of Adults With First‐Relapsed/Refractory Systemic Anaplastic Large‐Cell Lymphoma in the Pre‐Brentuximab Vedotin Era: A LYSA/SFGM‐TC Study,” European Journal of Cancer 83 (2017): 146–153. 28735072 10.1016/j.ejca.2017.06.026

[155] B. Pro , R. Advani , P. Brice , et al., “Brentuximab Vedotin (SGN‐35) in Patients With Relapsed or Refractory Systemic Anaplastic Large‐Cell Lymphoma: Results of a Phase II Study,” Journal of Clinical Oncology 30, no. 18 (2012): 2190–2196. 22614995 10.1200/JCO.2011.38.0402

[156] B. Pro , R. Advani , P. Brice , et al., “Five‐Year Results of Brentuximab Vedotin in Patients With Relapsed or Refractory Systemic Anaplastic Large Cell Lymphoma,” Blood 130, no. 25 (2017): 2709–2717. 28974506 10.1182/blood-2017-05-780049 PMC5746164

[157] A. Broccoli , C. Pellegrini , A. Di Rocco , et al., “Italian Real‐Life Experience With Brentuximab Vedotin: Results of a Large Observational Study of 40 Cases of Relapsed/Refractory Systemic Anaplastic Large Cell Lymphoma,” Haematologica 102, no. 11 (2017): 1931–1935. 28775121 10.3324/haematol.2017.171355 PMC5664397

[158] S. M. Horwitz , R. H. Advani , N. L. Bartlett , et al., “Objective Responses in Relapsed T‐Cell Lymphomas With Single‐Agent Brentuximab Vedotin,” Blood 123, no. 20 (2014): 3095–3100. 24652992 10.1182/blood-2013-12-542142 PMC4425442

[159] R. Aubrais , K. Bouabdallah , L. Chartier , et al., “Salvage Therapy With Brentuximab‐Vedotin and Bendamustine for Patients With R/R PTCL: A Retrospective Study From the LYSA Group,” Blood Advances 7, no. 19 (2023): 5733–5742. 36477770 10.1182/bloodadvances.2022008524 PMC10539874

[160] C. Gentille , H. Sarfraz , J. Joshi , J. Randhawa , S. Shah , and S. R. Pingali , “Use of Ifosfamide, Carboplatin and Etoposide in Combination With Brentuximab Vedotin or Romidepsin Based on CD30 Positivity in Relapsed/Refractory Peripheral T‐Cell Lymphoma,” Cancer Report 5, no. 7 (2022): e1581. 10.1002/cnr2.1581 PMC9327648 35263030

[161] P. L. Zinzani , G. Salles , A. J. Moskowitz , et al., “Nivolumab Plus Brentuximab Vedotin for Relapsed/Refractory Peripheral T‐Cell Lymphoma and Cutaneous T‐Cell Lymphoma,” Blood Advances 8, no. 10 (2024): 2400–2404. 38531062 10.1182/bloodadvances.2023011030 PMC11112622

[162] E. D. Jacobsen , J. P. Sharman , Y. Oki , et al., “Brentuximab Vedotin Demonstrates Objective Responses in a Phase 2 Study of Relapsed/Refractory DLBCL With Variable CD30 Expression,” Blood 125, no. 9 (2015): 1394–1402. 25573987 10.1182/blood-2014-09-598763

[163] J. P. Ward , M. M. Berrien‐Elliott , F. Gomez , et al., “Phase 1/Dose Expansion Trial of Brentuximab Vedotin and Lenalidomide in Relapsed or Refractory Diffuse Large B‐Cell Lymphoma,” Blood 139, no. 13 (2022): 1999–2010. 34780623 10.1182/blood.2021011894 PMC8972094

[164] P. L. Zinzani , A. Santoro , G. Gritti , et al., “Nivolumab Combined With Brentuximab Vedotin for Relapsed/Refractory Primary Mediastinal Large B‐Cell Lymphoma: Efficacy and Safety From the Phase II CheckMate 436 Study,” Journal of Clinical Oncology 37, no. 33 (2019): 3081–3089. 31398081 10.1200/JCO.19.01492 PMC6864847

[165] J. H. Park , I. Riviere , M. Gonen , et al., “Long‐Term Follow‐Up of CD19 CAR Therapy in Acute Lymphoblastic Leukemia,” New England Journal of Medicine 378, no. 5 (2018): 449–459. 29385376 10.1056/NEJMoa1709919 PMC6637939

[166] S. J. Schuster , J. Svoboda , E. A. Chong , et al., “Chimeric Antigen Receptor T Cells in Refractory B‐Cell Lymphomas,” New England Journal of Medicine 377, no. 26 (2017): 2545–2554. 29226764 10.1056/NEJMoa1708566 PMC5788566

[167] M. Katsin , D. Dormeshkin , A. Meleshko , A. Migas , S. Dubovik , and N. Konoplya , “CAR‐T Cell Therapy for Classical Hodgkin Lymphoma,” Hema 7, no. 12 (2023): e971. 10.1097/HS9.0000000000000971 PMC10656097 38026793

[168] C. M. Wang , Z. Q. Wu , Y. Wang , et al., “Autologous T Cells Expressing CD30 Chimeric Antigen Receptors for Relapsed or Refractory Hodgkin Lymphoma: An Open‐Label Phase I Trial,” Clinical Cancer Research 23, no. 5 (2017): 1156–1166. 27582488 10.1158/1078-0432.CCR-16-1365

[169] C. A. Ramos , B. Ballard , H. Zhang , et al., “Clinical and Immunological Responses After CD30‐Specific Chimeric Antigen Receptor‐Redirected Lymphocytes,” Journal of Clinical Investigation 127, no. 9 (2017): 3462–3471. 28805662 10.1172/JCI94306 PMC5669573

[170] J. N. Brudno , D. A. Natrakul , J. Karrs , et al., “Transient Responses and Significant Toxicities of Anti‐CD30 CAR T Cells for CD30+ Lymphomas: Results of a Phase 1 Trial,” Blood Advances 8, no. 3 (2024): 802–814. 37939262 10.1182/bloodadvances.2023011470 PMC10874855

[171] C. A. Ramos , N. S. Grover , A. W. Beaven , et al., “Anti‐CD30 CAR‐T Cell Therapy in Relapsed and Refractory Hodgkin Lymphoma,” Journal of Clinical Oncology 38, no. 32 (2020): 3794–3804. 32701411 10.1200/JCO.20.01342 PMC7655020

[172] S. Ahmed , I. W. Flinn , M. Mei , et al., “Updated Results and Correlative Analysis: Autologous CD30.CAR‐T Cells Therapy in Patients With Relapsed or Refractory Classical Hodgkin Lymphoma (CHARIOT Trial),” Blood 140 (2022): 7496–7497.

[173] A. C. Caballero Gonzalez , L. Escriba‐Garcia , R. Montserrat , et al., “Phase 1 Clinical Trial of Memory‐Enriched Academic HSP‐CAR30 for the Treatment of Relapsed/Refractory Hodgkin Lymphoma and CD30+ T‐Cell Lymphoma,” Blood 140 (2022): 405–407.

[174] D. Reef , C. J. Cheng , C. Babinec , et al., “CD30 CAR‐T in Relapsed and Refractory CD30+ Lymphomas. Long‐Term Follow‐Up of a Phase IB/II Clinical Trial,” Hematology and Oncology 43 (2025): 178–179.

[175] P. Zhang , X. Yang , Y. Cao , et al., “Autologous Stem Cell Transplantation in Tandem With Anti‐CD30 CAR T‐Cell Infusion in Relapsed/Refractory CD30(+) Lymphoma,” Experimental Hematology & Oncology 11, no. 1 (2022): 72. 36253833 10.1186/s40164-022-00323-9 PMC9578248

[176] D. Wang , C. Zeng , B. Xu , et al., “Anti‐CD30 Chimeric Antigen Receptor T Cell Therapy for Relapsed/Refractory CD30(+) Lymphoma Patients,” Blood Cancer Journal 10, no. 1 (2020): 8. 31974371 10.1038/s41408-020-0274-9 PMC6978321

[177] H. Karlsson , E. Svensson , C. Gigg , et al., “Evaluation of Intracellular Signaling Downstream Chimeric Antigen Receptors,” PLoS One 10, no. 12 (2015): e0144787. 26700307 10.1371/journal.pone.0144787 PMC4689545

[178] C. A. Ramos , R. Rouce , C. S. Robertson , et al., “In Vivo Fate and Activity of Second‐ Versus Third‐Generation CD19‐Specific CAR‐T Cells in B Cell Non‐Hodgkin's Lymphomas,” Molecular Therapy 26, no. 12 (2018): 2727–2737. 30309819 10.1016/j.ymthe.2018.09.009 PMC6277484

[179] N. S. Grover , A. Ivanova , D. T. Moore , et al., “CD30‐Directed CAR‐T Cells Co‐Expressing CCR4 in Relapsed/Refractory Hodgkin Lymphoma and CD30+ Cutaneous T Cell Lymphoma,” Blood 138 (2021): 742.

[180] N. S. Grover , G. Hucks , M. L. Riches , et al., “Anti‐CD30 CAR T Cells as Consolidation After Autologous Haematopoietic Stem‐Cell Transplantation in Patients With High‐Risk CD30(+) Lymphoma: A Phase 1 Study,” Lancet Haematology 11, no. 5 (2024): e358–e367. 38555923 10.1016/S2352-3026(24)00064-4 PMC11238265

[181] T. J. Voorhees , B. Zhao , J. Oldan , et al., “Pretherapy Metabolic Tumor Volume Is Associated With Response to CD30 CAR T Cells in Hodgkin Lymphoma,” Blood Advances 6, no. 4 (2022): 1255–1263. 34666347 10.1182/bloodadvances.2021005385 PMC8864661

[182] F. Meng , M. Xiang , Y. Liu , and D. Zeng , “Safety and Efficacy of Anti‐CD30 CAR‐T Cell Therapy in Relapsed/Refractory Classic Hodgkin Lymphoma: A Systematic Review and Meta‐Analysis,” BMC Cancer 25, no. 1 (2025): 78. 39806291 10.1186/s12885-024-13400-5 PMC11731380

[183] M. Mei , S. Ahmed , A. Beitinjaneh , et al., “Phase 1b/2 Study of Autologous CD30.CAR‐T Cells in Combination With Nivolumab in Patients With Relapsed or Refractory Hodgkin Lymphoma After Failure of Frontline Therapy (ACTION),” Blood 140 (2022): 12728–30.

[184] D. H. Kim and F. Vega , “Relapsed Classic Hodgkin Lymphoma With Decreased CD30 Expression After Brentuximab and Anti‐CD30 CAR‐T Therapies,” Blood 139, no. 6 (2022): 951. 35142847 10.1182/blood.2021013881

[185] M. L. Marques‐Piubelli , D. H. Kim , L. J. Medeiros , et al., “CD30 Expression Is Frequently Decreased in Relapsed Classic Hodgkin Lymphoma After Anti‐CD30 CAR T‐Cell Therapy,” Histopathology 83, no. 1 (2023): 143–148. 36994939 10.1111/his.14910

[186] M. L. Marques‐Piubelli , D. H. Kim , and L. J. Medeiros , “Expression of Concern: CD30 Expression Is Frequently Decreased in Relapsed Classic Hodgkin Lymphoma After Anti‐CD30 CAR T‐Cell Therapy,” Histopathology 84, no. 3 (2024): 574. 38155436 10.1111/his.15124

[187] N. Martarelli , M. Capurro , G. Mansour , et al., “Artificial Intelligence‐Powered Molecular Docking and Steered Molecular Dynamics for Accurate scFv Selection of Anti‐CD30 Chimeric Antigen Receptors,” International Journal of Molecular Sciences 25, no. 13 (2024): 7231. 39000338 10.3390/ijms25137231 PMC11242627

[188] N. Martarelli , M. Capurro , A. Scialdone , et al., “Dual Targeting CD30/PDL1 CAR‐T Cells Outperforms CD30 CAR T Cells in a Newly Developed Immunosuppresssive 2D Cellular Model Mimicking Classic HL Microenvironment,” Hematological Oncology 43 (2025): 149–150.

[189] W. Sang , X. Wang , H. Geng , et al., “Anti‐PD‐1 Therapy Enhances the Efficacy of CD30‐Directed Chimeric Antigen Receptor T Cell Therapy in Patients With Relapsed/Refractory CD30+ Lymphoma,” Frontiers in Immunology 13 (2022): 858021. 35432352 10.3389/fimmu.2022.858021 PMC9010867

[190] D. Jagadeesh , S. Horwitz , N. L. Bartlett , et al., “Response to Brentuximab Vedotin by CD30 Expression in Non‐Hodgkin Lymphoma,” Oncologist 27, no. 10 (2022): 864–873. 35948003 10.1093/oncolo/oyac137 PMC9526494

[191] W. Chen and Z. Zhang , “Recent Advances in Understanding the Clinical Responses of Brentuximab Vedotin in Lymphoma and the Correlation With CD30 Expression,” Oncotargets and Therapy 18 (2025): 1–14. 39802262 10.2147/OTT.S487088 PMC11720807

[192] Y. H. Kim , M. Tavallaee , U. Sundram , et al., “Phase II Investigator‐Initiated Study of Brentuximab Vedotin in Mycosis Fungoides and Sezary Syndrome With Variable CD30 Expression Level: A Multi‐Institution Collaborative Project,” Journal of Clinical Oncology 33, no. 32 (2015): 3750–3758. 26195720 10.1200/JCO.2014.60.3969 PMC5089160

[193] A. H. Staudacher and M. P. Brown , “Antibody Drug Conjugates and Bystander Killing: Is Antigen‐Dependent Internalisation Required?,” British Journal of Cancer 117, no. 12 (2017): 1736–1742. 29065110 10.1038/bjc.2017.367 PMC5729478

